# Differential association of primary and alternative σ factors with RNA polymerase of *Mycobacterium tuberculosis* during transcription elongation

**DOI:** 10.1093/nar/gkaf1459

**Published:** 2026-01-08

**Authors:** Nilanjana Hazra, Jayanta Mukhopadhyay

**Affiliations:** Department of Chemical Sciences, Bose Institute, EN 80, Sector V, Bidhan Nagar, Kolkata 700091, India; Department of Chemical Sciences, Bose Institute, EN 80, Sector V, Bidhan Nagar, Kolkata 700091, India

## Abstract

In response to environmental cues, bacteria alter gene expression by switching σ factor(s) that recognize a specific set of promoters. This ‘σ-cycle’, in which σ binds to RNA polymerase (RNAP) to initiate transcription and is released during transcription elongation, is thought to be an essential step for σ-switching. Studies in *Escherichia coli* provide evidence that σ^70^ is stochastically released from the elongation complex. This ‘σ-cycle’ became a general model for all σ factors of all bacteria. Here, using three distinct groups of σ factors in *Mycobacterium tuberculosis*, we show that although a significant fraction of σ^A^ and σ^E^ are stochastically or immediately released from RNAP during the transition from transcription initiation to elongation, most of σ^F^ are retained. We also show that NusA facilitates σ^A^ release from elongation complexes at specific genes but does not affect the release of σ^E^ and σ^F^. Our results further indicate that σ^F^ remains bound at the elongation complex despite the displacement of σ_4_, as σ_2_ and σ_3_ remain associated with RNAP. Our study demonstrates that σ-release is not a universal phenomenon—the release or retention of σ factor depends on its domain architecture.

## Graphical Abstract

**Graphical Abstract:**
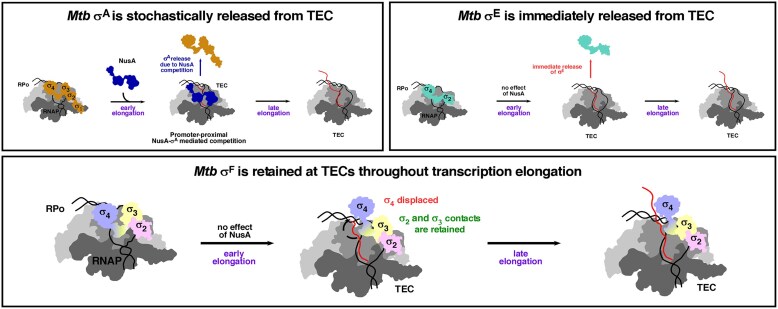

## Introduction

Transcription is the first step to gene regulation in bacteria. All environmental stress signals relayed down by the cell converge at the point of transcription to elicit a wave of altered gene expression for survival. The altered gene expression is achieved by replacing the primary σ factor with one or more alternative σ factor(s) [1–3]. The ‘σ cycle’ in which σ binds to RNAP core (α2ββ’ω) to initiate transcription and releases stochastically or obligatorily from RNAP during transcription elongation is thought to be an essential step for ‘σ-switching’ [4–9]. The σ release indicates that the affinity of σ is reduced in the transcription elongation complex (TEC) compared to the initiation complex (RPitc) [6]. Notably, the primary σ (including σ^70^) contains four conserved regions or domains, all of which interact with RNAP core to form RNAP holoenzyme [10–13]. In the RNAP-promoter open complex (RPo), the σ regions 4 and 2 interact with the promoter elements −35 and −10, respectively, whereas σ_3_/σ_4_ linker region occupies the RNA exit channel, and σ region 1.1 stays out of the active-centre cleft [14]. Once RNAP synthesizes more than ∼11 nt long RNA, the nascent elongating RNA displaces the σ_3_/σ_4_ linker, which triggers the expulsion of σ region 4 from the promoter and RNAP [15–19]. Thus, the structural transition of the RNAP-DNA-RNA ternary complex during the transition from transcription initiation to elongation accounts for the reduced affinity of σ and could explain the immediate or stochastic release of σ upon TEC formation. There are also evidences that a certain fraction of σ remains associated with the RNAP throughout the transcription cycle [20–22]. Additional evidence of reduced affinity of σ to RNAP core at TEC is due to the σ-NusA competition, where the elongation factor NusA outcompetes σ to bind the TEC and could be a part of the ‘σ cycle’ [6, 23, 24]. It is important to note that all the studies were performed in *Escherichia coli (Eco)*, but σ-release is considered a general phenomenon for all σ factors across the bacterial species. Our recent studies demonstrated that *Bacillus subtilis* σ^A^ (having a smaller σ_1.1_ and a different architecture than σ_1.1_ in *E. coli*) and an *E. coli* σ^70^ variant lacking σ_1.1_ remain stably associated with transcription complexes [25]. This is in stark contrast to full-length *E. coli* σ^70^, which is released stochastically during elongation. This observation indicates, σ_1.1_ plays a critical role in its release or retention in TEC.

**Figure 1.**
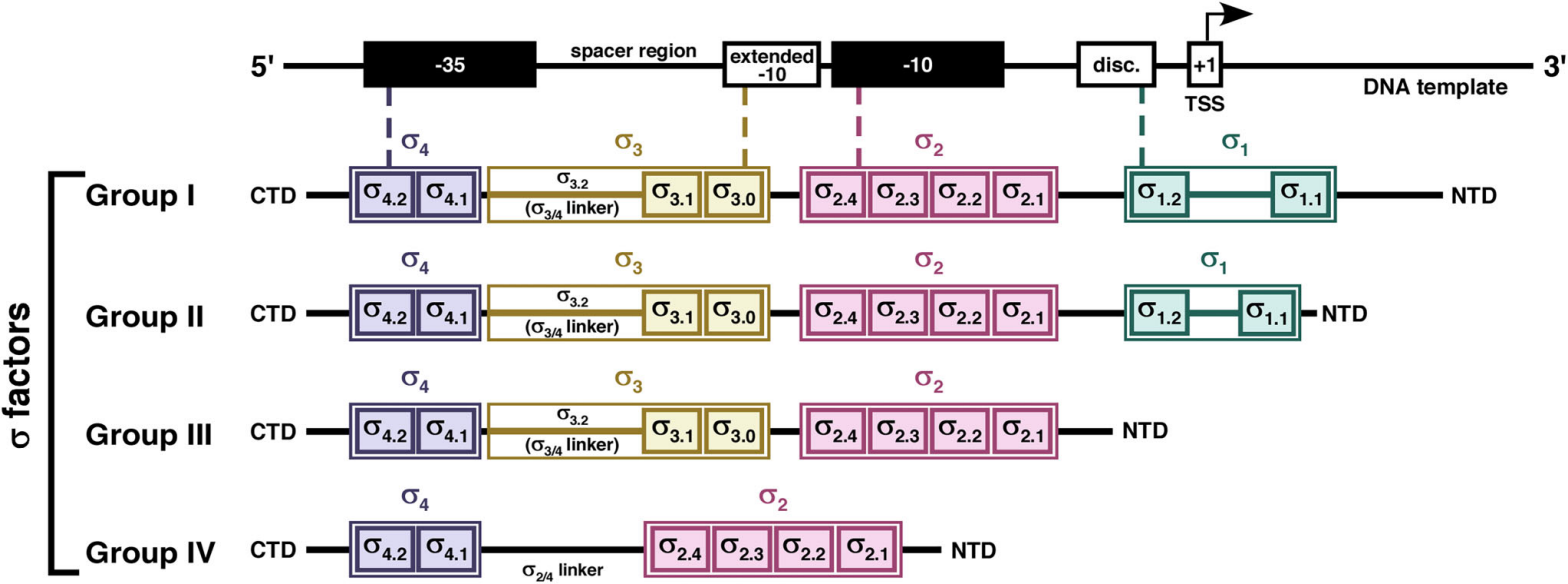
Schematic representation of *Mtb* σ factor groups. σ factors are categorized into four groups based on domain/region composition (major domains σ_1_, σ_2_, σ_3_, and σ_4_). The group I σ factor (principal σ, σ^A^) comprises all four domains with a long N-terminal tail. The group II σ factor (σ^B^) consists of all four major domains, with a shorter N-terminal chain. The group III σ factor (σ^F^) comprises σ_2_, σ_3_, and σ_4_, and group IV σ factors (σ^C^–σ^E^, σ^G^–σ^M^) comprise only σ_2_ and σ_4_ joined by a flexible linker. The σ domains 1.2, 2, 3, and 4 recognize the promoter discriminator element (disc.), −10 motif, extended −10 motif, and −35 motif, respectively.

*Mycobacterium tuberculosis (Mtb)* contains 13 σ factors that belong to four groups comprising various conserved regions (Fig. 1). The primary σ factor, σ^A^, responsible for the regulation of the housekeeping genes, belongs to group I σ factors [26, 27]. This group, like *Eco* σ^70^, comprises four conserved regions (σ_1.1_, σ_2_, σ_3_, and σ_4_) for promoter recognition and are joined by flexible linkers [10–13]. The alternative σ factors whose levels increase in response to specific stresses are categorized into group II (principal like σ factor, σ^B^), group III (σ^F^), and group IV (σ^C^, σ^D^ σ^E^, σ^G^, σ^H^, σ^I^, σ^J^, σ^K^, σ^L^, and σ^M^) [28]. The group III σ factors contain three of the four σ domains (σ_2_, σ_3_, and σ_4_) and require all three motifs (−35, extended −10 and −10 motif) for promoter recognition [29–31]. The group IV factors exhibiting the most abundant and diverse group of σs represent the ECF (extra-cytoplasmic function) family and contain only two domains (σ_2_ and σ_4_) for −10 and −35 element recognition [32, 33].

Since σ-conserved regions may play an important part in the σ-release or retention in the TEC, we have tested the nature of σ-release for the representative σ factors (σ^A^, σ^E^, σ^F^) from each of the three distinct structural groups. We have observed differential retention of three σ factors σ^A^, σ^E^, and σ^F^ during transcription elongation. The *in vitro* assays have indicated that σ^A^ is stochastically released during elongation. While CarD and RbpA that promote transcription initiation in *M. tuberculosis* promoter [34–36], do not affect the σ^A^ release from TEC, the extent of σ^A^ release is significantly enhanced in the presence of NusA at certain genes. However, NusA does not have any effect on the σ-release for the σ^E^ and σ^F^. A significant fraction of the σ^E^ is released immediately upon the transition from transcription initiation to elongation. In contrast, most of the σ^F^ remains associated with the TEC. The *in vivo* assays corroborate our *in vitro* observations—*Mtb* σ^F^ is thoroughly retained with the TEC throughout the transcription process, while *Mtb* σ^A^ and σ^E^ demonstrate a varying degree of gradual or stochastic release from the TECs.

The fluorescence resonance energy transfer (FRET) assay between three conserved regions of σ^F^ and RNAP further reveals a displacement or rearrangement of the σ_4_ in TEC from its initial position in RPo, while the positions of σ_2_ and σ_3_ remain unaltered in the TEC. Subsequently, we observed that the affinity of σ to TEC is significantly reduced for σ^A^ and σ^E^ compared to its affinity to RNAP core. In contrast, although the affinity of σ^F^ for the TEC was lower than for the core enzyme, it remained sufficiently strong to allow stable association with the TEC.

Collectively, this study revises the long-held assumption of a universal σ-cycle, suggesting that bacteria have evolved a repertoire of mechanisms fine-tuned for context-specific gene expression. The differential release and retention of σ factors may thus represent an evolutionary adaptation, possibly allowing mycobacteria to orchestrate robust survival strategies in response to the diverse challenges of environmental and host-mediated stress.

## Materials and methods

### Cloning strategies

The plasmids used for purification of wildtype *Mtb* RNAP core were existing pET Duet-*rpoB*-*rpoC* and pCOLA Duet-*rpoA*-*rpoZ* (subcloned from existing pACYC Duet-*rpoA*-*rpoZ* using NcoI-KpnI) [37]. RNAP core derivative was purified using pEVOL-pAzF (a kind gift from Peter Schultz), mutated pET Duet-*rpoB*-*rpoC*, and pCOLA Duet-*rpoA*-*rpoZ* (Supplementary Table S1) plasmids [38]. *Mtb* σ^A^ was subcloned from existing pACYC Duet-*rpoA*-*σ^A^* to pET Duet-1 using restriction sites EcoRI-NotI. *Mtb* σ^E^ and σ^F^ were purified using plasmids pET30a-*σ^E^* and pET30a-*σ^F^*, respectively [39]. The *nusA* gene was amplified from *Mtb* genomic DNA using primers listed in Supplementary Table S2 and cloned into pACYC Duet-1 vector with restriction sites EcoRI-HindIII. pET28a-*rbpA* plasmid was used for *Mtb* RbpA purification. The c*arD* genewas amplified from *Mtb* genomic DNA and cloned into pET Duet-1 plasmid using BamHI-KpnI sites. *Mtb* σ^A^ and σ^E^ genes were amplified from genomic DNA using primers listed in Supplementary Table S2 and cloned into pLAM12 vector with sites NdeI-HindII and NdeI-EcoRI, respectively. *Mtb* σ^F^ was subloned into pLAM12 from pET30a-*σ^F^* using NdeI-BamHI.

### Unnatural amino acid mutagenesis, purification and labelling of *Mtb* RNAP core derivative

Amber codon (TAG) was inserted into *rpoC* (β’) subunit of RNAP by site-directed mutagenesis at 359th codon (D359Amber) in pET Duet-*rpoB*-*rpoC* plasmid using primers listed in Supplementary Table S3 (Supplementary Fig. S1A–C). The natural TAG (amber) stop-codon of genes *rpoC, rpoA*, and *rpoZ* were mutated to TAA (ochre) using site-directed mutagenesis. All mutations were confirmed by sequencing.

*Escherichia coli* BL21 (DE3) cells were co-transformed with the plasmid constructs and pEVOL-pAzF plasmid. Colonies were selected using three antibiotics (100 µg/ml ampicillin, 35 µg/ml chloramphenicol, and 50 µg/ml kanamycin). A single colony was transferred to 10 ml 2XYT (16 g tryptone,10 g yeast extract, 5 g NaCl, pH 7.5 in 1 l) broth containing respective antibiotics and grown for 16 h at 37°C with shaking. One millilitre inoculum was transferred to 1 l 2XYT media with respective antibiotics, and cells were grown at 37°C with shaking till O.D_600_ reached 0.4. The culture temperature was brought down to 16°C before 0.5 mM isopropyl β-D-1-thiogalactopyranoside (IPTG) induction.One millimolar 4-azido-L-phenylalanine (dissolved in 1 N NaOH) and 1 ml of 20% arabinose were added to the culture during induction. The culture was grown at 16°C for 16 h in the dark with shaking. The cells were pelleted down by centrifugation at 5000 rpm at 4°C for 10 min. The cell pellet was resuspended in lysis buffer (20 mM Tris–HCl, pH 7.9, 0.2 M NaCl, 5% glycerol, 0.025% sodium deoxycholate, 1 mM phenylmethylsulfonyl fluoride or PMSF, and 1 mM dithiothreitol or DTT) and lysed using UP100H sonicator with seven pulses of 30 s each (with intermittent rest in ice) at 80% sonicator output. The sonicated sample was centrifuged at 13 000 rpm at 4°C for 30 min. The supernatant was treated with 0.5% polymin-P and kept in ice for 20 min, followed by a spin at 13 000 rpm at 4°C for 30 min. The pellet was washed with TG buffer containing 20 mM Tris–HCl, pH 7.9, 0.5 M NaCl, and 5% glycerol, kept on ice for 30 min, followed by centrifugation at 13 000 rpm at 4°C for 30 min. The pellet obtained was washed with TG buffer containing 20 mM Tris–HCl, pH 7.9, 1 M NaCl and 5% glycerol, kept in ice for 45 min, followed by centrifugation at 13 000 rpm at 4°C for 30 min. The supernatant obtained was treated with 25% ammonium sulphate and centrifuged at 13 000 rpm at 4°C for 30 min. The pellet obtained was dissolved in TG buffer containing 20 mM Tris–HCl, pH 7.9, 0.2 M NaCl, and 5% glycerol, followed by purification with Ni-NTA affinity chromatography. RNAP derivative was successfully eluted with 80 mM imidazole. The protein was further purified by ion-exchange chromatography using FPLC (Fast Protein Liquid Chromatography). The purified protein was concentrated using 100 kDa concentrator and quantified.

For labelling of the purified RNAP derivative, Dylight488-phosphine was dissolved in dimethyl sulfoxide or DMSO and quantified. RNAP core derivative was labelled by Staudinger–Bertozzi ligation reaction with Dylight-488-phosphine conjugate dye (ratio 1:5) at 4°C for 48 h in dark [40]. Excess dye was removed using Biogel-P6 (Bio-Rad Inc) desalting column. A labelling efficiency of 71% was achieved for the labelled RNAP core derivative. The labelled RNAP core derivative was analysed using 10% sodium dodecyl sulphate–polyacrylamide gel electrophoresis (SDS–PAGE) and visualized using fluorescence scanning (Dylight488 channel) by Amersham Typhoon Imager, GE Healthcare (Supplementary Fig. S1C). The activity of the labelled RNAP was confirmed by *in vitro* transcription (Supplementary Fig. S1E).

The wildtype RNAP core protein was purified using a procedure similar to the RNAP core derivative. *Escherichia coli* BL21 (DE3) cells were co-transformed with plasmids pET Duet-*rpoB*-*rpoC* and pCOLA Duet-*rpoA*-*rpoZ* and grown under selection of two antibiotics (100 µg/ml ampicillin and 50 µg/ml kanamycin) [37]. The 4-azido-L-phenylalanine and arabinose were not used during induction. The protein was resolved using 10% SDS–PAGE and quantified.

### Purification and labelling of *Mtb* σ subunits

*Escherichia coli* BL21 (DE3) cells were transformed with pETDuet-*σ^A^*, pET30a-*σ^F^*, or pET30a-*σ^E^* for purification of *Mtb* σ^A^, σ^F^, and σ^E^_,_ respectively [39]. The colonies obtained were selected for antibiotics (100 µg/ml ampicillin for pET Duet-*σ^A^*; 50 µg/ml kanamycin for pET30a-*σ^F^* and pET30a-*σ^E^* transformed cells).

A single colony was transferred to 10 ml 2XYT broth supplied with respective antibiotics and grown for 16 h at 37°C with shaking. One millilitre of inoculum was transferred to 1 l 2XYT broth supplied with the respective antibiotic and incubated at 37°C with shaking till O.D_600_ reached 0.4. The culture temperature was cooled to 16°C followed by 0.2 mM IPTG induction. The cultures were grown at 16°C for 16 h with shaking. The cells were centrifuged at 5000 rpm for 10 min. The cell pellet was resuspended with TG buffer containing 40 mM Tris–HCl, pH 7.9, 0.2 M NaCl, and 5% glycerol for σ^A^ and 40 mM Tris–HCl, pH 7.9, 0.4 M NaCl, and 5% glycerol for σ^F^ and σ^E^. The cells were sonicated and centrifuged at 13 000 rpm for 30 min at 4°C. The supernatant was treated with 25% ammonium sulphate followed by centrifugation at 13 000 rpm at 4°C for 30 min. The obtained pellet was dissolved in the respective TG buffers mentioned above. The *his*-tagged proteins were purified using Ni-NTA affinity chromatography and eluted with 250 mM imidazole. The σ factors were further purified by ion-exchange chromatography using FPLC. The purified proteins were then concentrated using 10 kDa concentrators and their purities were analysed using 10% SDS–PAGE (Supplementary Fig. S1D).

For the labelling of the purified σ factors, the protein samples were treated with 10 mM DTT for 72 h to reduce the disulfide bonds between cysteine residues. DTT was removed from the samples by 60% ammonium sulphate precipitation followed by centrifugation at 13 000 rpm for 30 min at 4°C. The pellet obtained was dissolved in labelling buffer [100 mM Na_2_PO_4_, pH 7.5, 200 mM NaCl, and 1 mM ethylenediaminetetraacetic acid (EDTA)]. The protein and tetramethylrhodamine-maleimide (TMR) or Alexafluor-488 dye were added in a 1:5 ratio followed by 3 h of incubation in ice. The excess dye was removed using Biogel-P6 desalting column purification. Labelling efficiency of the TMR and Alexafluor-488 labelled samples was ∼85% and ∼98%, respectively. The activity of labelled proteins was confirmed by *in vitro* transcription (Supplementary Fig. S1F–H).

### Purification of *Mtb* NusA, RbpA, and CarD proteins

As detailed in the previous section, *Mtb* NusA, RbpA, and CarD proteins were purified using the same protocol as σ^F^ or σ^E^, except pACYC Duet–1 *nusA*, pET−28a *rbpA*,and pET Duet–1 *carD* plasmids were individually used to transform the *Escherichia coli BL21* (DE3) cells under chloramphenicol (35 µg/ml), kanamycin (50 µg/ml), and ampicillin (100 µg/ml) selection, respectively. The purified proteins were concentrated using 10 kDa concentrators, and their purities were analysed using 10% SDS–PAGE (Supplementary Fig. S1D).

### Site-directed mutagenesis, purification and labelling of σ^A^, σ^F^, and σ^E^ derivatives

The single cysteine residue of *Mtb* σ^A^ was substituted with an alanine residue by site-directed mutagenesis, and new cysteine residues were introduced at 331, 400, and 472 positions individually to label σ_2_, σ_3_, and σ_4_ domains, using primers listed in Supplementary Table S3. The cysteine residue of wildtype σ^F^ (positioned at σ_2_) was substituted with alanine by site-directed mutagenesis, and cysteine residues were incorporated at position 156 to label the protein at the σ_3_ domain and at position 235 to label the σ_4_ domain, respectively. The σ^E^ constructs containing a single cysteine residue were prepared by alanine substitution—the σ^E^ domain 2 was labelled at the 92nd residue, and domain 4 was labelled at the 204th residue (natural cysteine). The single cysteine-containing proteins at respective regions (σ_2_, σ_3_, and σ_4_ for σ^A^ and σ^F^; σ_2_ and σ_4_ for σ^E^) were tagged with TMR-maleimide and subsequently used for *in vitro* FRET assays.

### Labelling and purification of DNA fragments

Long oligonucleotides containing promoter and promoter downstream sequences were obtained from Integrated DNA Technologies Ltd. (IDT) and annealed to form double-stranded DNA. These DNA templates were used directly in the experiments or were amplified with specific primer pairs to obtain longer fragments (Supplementary Tables S4 and S5). Each promoter DNA derivative had a specific nucleotide appearing first after a certain sequence length downstream of the transcription start site (TSS). σ^A^-specific promoters *sinP3* had first C appearing at +17, +24, +30, +60; *rrnAP3* had first A appearing at +11, +17, +25, and *sigA* template had first T appearing at +17, and +30. σ^F^-specific promoters *usfXP1* and *phoY1* had first T at +18, +24, +29, +65 and A at +18, +29, respectively. σ^E^-specific promoters *sigB* and *hsp20* had first T appearing at +16, +24, +28, +85, and +16, +29, respectively. The sequences of all DNA fragments used in the experiments are listed in Supplementary Table S4. Respective DNA fragments were polymerase chain reaction (PCR) amplified by Cy5-tagged primers ordered from IDT and polyacrylamide gel electrophoresis or PAGE purified (Supplementary Table S6). Alternatively, some DNA templates were labelled in-gel during electrophoretic mobility shift assay (EMSA) assays using the SYBR Gold nucleic acid stain in TAE buffer (40 mM Tris–HCl, 20 mM acetic acid, and 1 mM EDTA). The choice of the fluorescent probe (Cy5 or SYBR Gold) was made according to the probe availability during the experiments or to ensure optimum DNA labelling.

### Electrophoretic mobility shift assay

75 nM RNAP core and 75 nM labelled σ were incubated in 10 μl of transcription buffer (45 mM Tris–HCl, pH 8.0, 5 mM MgCl_2_, 70 mM KCl, 1.5 mM MnCl_2_, 10% glycerol, and 1 mM DTT) at 25°C for 5 min to form holoenzyme. Twenty-five nanomolar 5′ Cy5-tagged DNA was added and incubated for 10 min at 37°C to form RPo. Stalled TEC_+n_ was formed by the addition of 3 NTPs and 25 ng/µl heparin at 37°C for 10 min, with the NTPs having a final working concentration of 125 µM, excluding the 4th NTP, which is responsible for pausing RNAP at the nth position. The reaction was resolved using 5% native PAGE in Tris-Borate-EDTA or TBE (89 mM Tris base, 89 mM boric acid, 2 mM EDTA) running buffer at 4°C. The gels were scanned for fluorescence by Amersham Typhoon, GE Healthcare using 488 (for Alexafluor-488 tagged σ), TMR (for TMR-maleimide tagged σs) and Cy5 (for 5′ Cy5 tagged DNA) channels. For reactions with *Mtb* NusA, labelled σ^A^ was incubated with RNAP core and promoter DNA to form RPo. Then, 150 nM labelled NusA was added, followed by 3 NTPs and heparin, and incubated for 10 min at 37°C, to stall RNAP at TEC_+n_. For reactions with initiation factors, 150 nM of RbpA/CarD were added with 75 nM holoenzyme and incubated at 37°C for 5 min. Twenty-five nanomolar DNA was added to form RPo at 37°C for 10 min. One hundred and twenty five micromolar each of the 3 NTPs and 25 ng/µl heparin were added, followed by incubation at 37°C for 10 min. The reactions were resolved using 5% native PAGE in TBE (89 mM Tris base, 89 mM boric acid, 2 mM EDTA) running buffer at 4°C. The gels were scanned for fluorescence by Amersham Typhoon, GE Healthcare.

For the transcription complexes that were formed using TMR-labelled σ, unlabelled RNAP and unlabelled DNA, the EMSA gels were first scanned with the TMR channel for σ detection. Next, the gels were stained with SYBR Gold nucleic acid stain to label the DNA in TAE buffer (40 mM Tris–HCl, 20 mM acetic acid, and 1 mM EDTA) for 20 min at 25°C. The SYBR Gold-stained gel was then scanned for fluorescence using 488 channel of Amersham Typhoon, GE Healthcare. In case of complexes with Alexafluor-488 tagged σ, the EMSA gels were first scanned with 488 channel for σ detection and then subsequently treated with SYBR Gold stain to label the DNA. The labelled DNA was detected using the Cy3 channel of the Amersham Typhoon Imager to avoid unwanted detection of 488 labelled σ. Note that due to the broad excitation/emission spectrum of the SYBR Gold dye, it can be successfully detected by both 488 and Cy3 channels with significant signal intensities, unlike the other dyes used in our experiments, which are typically detected only by their respective fluorescence channels.

The amount of the complex was determined by the ratio of the amount of DNA in the complex with respect to total DNA (DNA_bound _+ DNA_unbound_). The fractional occupancies of respective σ factors at RPo, RPitc and TEC were calculated as a ratio of the bound σ and complex. The amount of RPo was normalized to 1, and the fractional occupancies at RPitc and TECs were estimated accordingly. All the fractional occupancies were corrected for the subpopulation of RPo that are not competent to form TEC. The corrected fractional σ occupancies were calculated using the formula: E_TEC_ = [E_RPO+NTPs_ − (1 − f)E_RPo_]/f, where f is the transcription efficiency, i.e. the fraction of RPo complexes that form TEC, and E is the fractional occupancy of σ at RPo or after addition of NTPs. The σ occupancies were plotted against RPo, RPitc, and TECs as bar graphs. Each data set represents a mean of three replicates, and error bars represent the standard deviations. One-way ANOVA (Analysis of Variance) was performed for statistical significance testing against a *P*-value of .05 (*, **, ***, **** denotes *P *<.05, .01, .005, and .001, respectively; ns denotes nonsignificance).

### *In vitro* transcription assay

**Figure 2.**
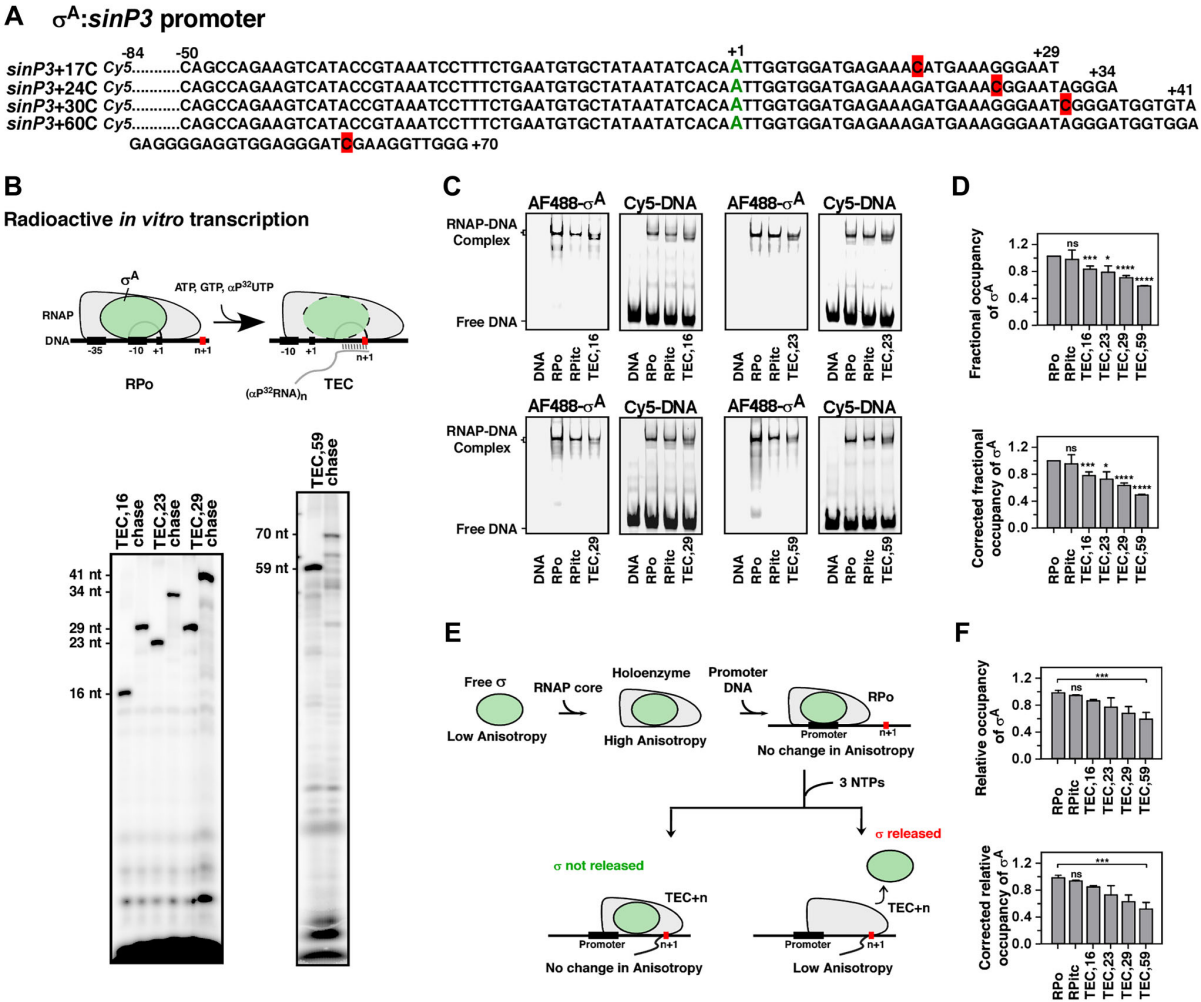
Fractional occupancies of *Mtb* σ^A^ in RPo and TECs *in vitro*: EMSA and fluorescence anisotropy assays. (**A**) Sequences of *sinP3* nontemplate DNA strands used to form TECs. The TSS (+1) are represented in green, and the RNAP pause sites are highlighted in red. (**B**) Formation of RNA transcripts by paused TECs by radioactive *in vitro* transcription (IVT) assay. **Upper**: Schematic representation of pause experiments to visualize radioactive αP^32^-tagged RNA formed by stalled TECs. **Lower**: RNAP and Alexafluor-488 (AF488) labelled σ^A^ were incubated with *sinP3* DNA template to form RPo. TEC + *n* (*n* = 16, 23, 29, and 59) were formed by adding NTPs (ATP, αP^32^-UTP, and GTP) and heparin. The stalled TECs form αP^32^-RNA transcripts of lengths 16, 23, 29, and 59 nt, respectively. Further addition of CTP rescues paused RNAP to form full-length αP^32^-RNA (chase). The transcripts were resolved using 15% (*n* = 16, 23, and 29) and 8% (*n* = 59) denaturing urea–PAGE. (**C**) RNAP and Alexafluor-488 (AF488) labelled σ^A^ were incubated with Cy5 labelled *sinP3* to form RPo. Tec + *n* (*n* = 16, 23, 29, and 59) were formed by adding NTPs (ATP, UTP, and GTP). The resulting complexes were resolved in 5% native PAGE. The gel was scanned by fluorescence imager (488 channel: left panel shows AF488 labelled σ^A^; Cy5 channel: right panel shows Cy5 labelled DNA). Retarded bands both in 488 and Cy5 channels were depicted as RPo, RPitc, or TEC. Two consecutive retarded bands were observed for TECs at +16, +23, +29, and +59, both retaining σ^A,^ indicating two sub-populations of TECs at *sinP3* with different conformations. (**D**)** Upper:** Fractional occupancies of σ^A^ at RPitc and TECs with respect to RPo, from EMSA. **Lower**: Fractional occupancies of σ^A^ after correction for the subpopulation of RNAP complexes at RPo, incompetent to form TECs at respective *n* + 1 sites. Each bar represents a mean of three independent replicates with standard deviations represented as error bars; statistical significance was assigned based on *P*-value <.05 (*, ***, **** denotes *P *< .05, .005, and .001, respectively; ns denotes nonsignificance). (**E**) Schematic representation of anisotropy assay to assess relative σ occupancies at TECs. Free σ in solution has the lowest anisotropy, which increases upon RPo formation. The addition of three NTPs to stall RNAP at elongation results in either no change in the anisotropy value for σ nonrelease or a decrease in the anisotropy value for σ release. (**F**)** Upper**: Relative occupancies of σ^A^ at RPitc and TEC_+n_ normalized with σ^A^ occupancy at RPo, from anisotropy assay. **Lower**: Relative occupancy of σ^A^ after correcting for the subpopulation of RNAP complexes at RPo, incompetent to form TECs. Each bar represents a mean of three independent replicates with standard deviations represented as error bars; statistical significance was assigned based on *P*-value <.05 (***denotes *P *<.005, ns denotes nonsignificance).

**Figure 3.**
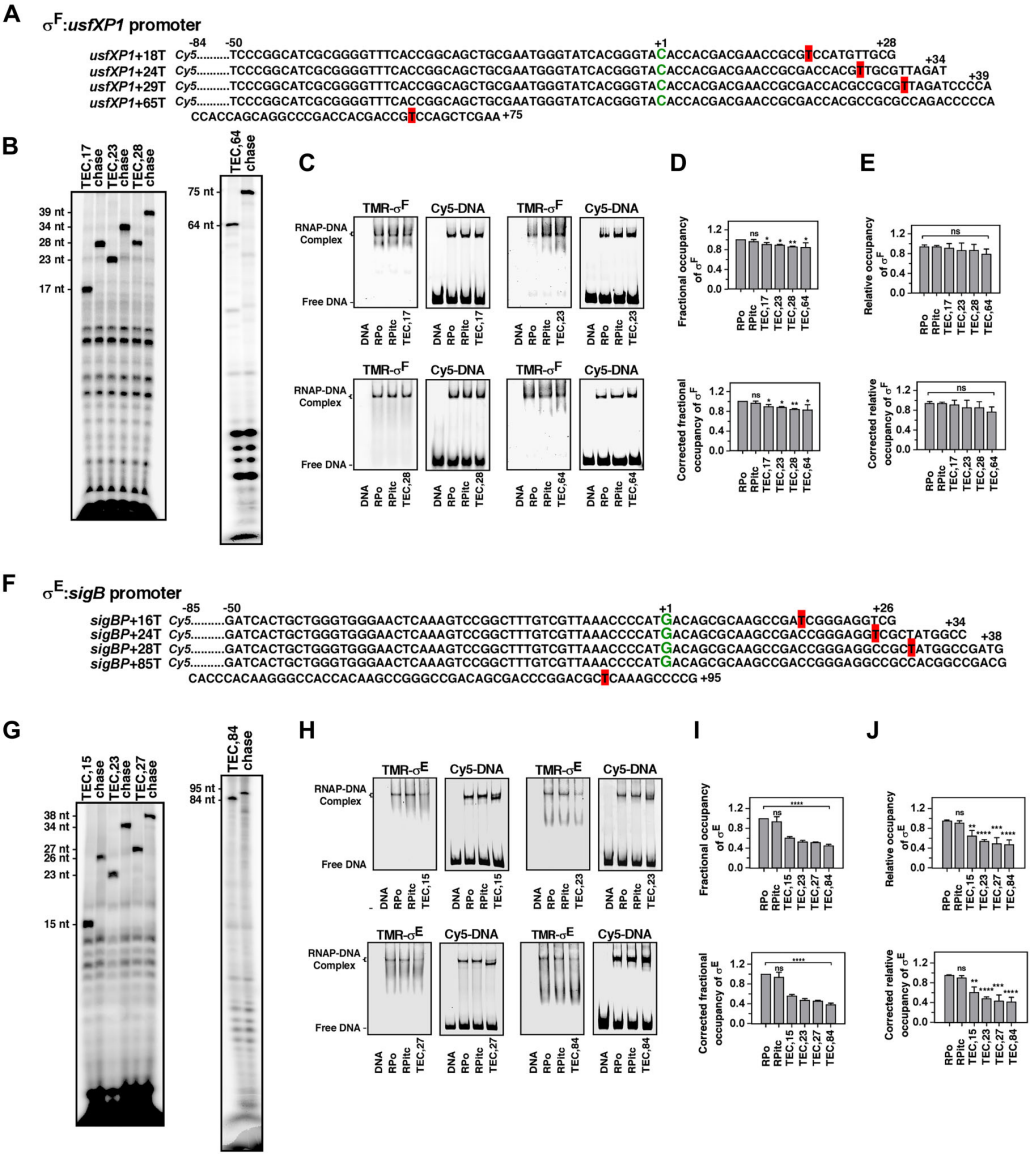
Fractional occupancies of *Mtb* σ^F^ and σ^E^ in RPo and TECs *in vitro*: EMSA and fluorescence anisotropy assays.(**A**) Sequences of *usfXP1* nontemplate DNA strands used to form TECs with σ^F^. The TSS (+1) are represented in green, and the RNAP pause sites are highlighted in red. (**B**) Formation of RNA transcripts by paused TECs by radioactive IVT assay on *usfXP1* promoter. (**C**) RNAP and TMR-labelled σ^F^ were incubated with Cy5-labelled *usfXP1* template to form RPo. TEC_+n_ (*n* = 17, 23, 28, and 64) were formed by adding NTPs (ATP, GTP, and CTP). The resulting complexes were resolved in 5% native PAGE. The gel was scanned by fluorescence imager (TMR channel: left panel shows TMR-labelled σ^F^; Cy5 channel: right panel shows Cy5-labelled DNA). (**D**) **Upper:** Fractional occupancies of σ^F^ at RPitc and TECs with respect to RPo, from EMSA. **Lower:** Fractional occupancies of σ^F^ after correction for the subpopulation of RNAP complexes at RPo, incompetent to form TECs at respective *n* + 1 sites. Each bar represents a mean of three independent replicates with standard deviations represented as error bars; statistical significance was assigned based on *P*-value <.05 (*, and ** denotes *P *<.05, and .01, respectively; ns denotes nonsignificance). (**E**) **Upper:** Relative occupancies of σ^F^ at RPitc and TEC_+n_ normalized with σ^F^ occupancy at RPo, from anisotropy assay. **Lower:** Relative occupancy of σ^F^ after correcting for the subpopulation of RNAP complexes at RPo, incompetent to form TECs. Each bar represents a mean of three independent replicates with standard deviations represented as error bars; statistical significance was assigned based on *P*-value <.05 (ns denotes nonsignificance). (**F**) Sequences of *sigB* nontemplate DNA strands used to form TECs with σ^E^. (**G**) Formation of RNA transcripts by paused TECs by radioactive IVT assay on *sigB* promoter by σ^E^. (**H**) RNAP and TMR-labelled σ^E^ were incubated with Cy5-labelled *sigB* template to form RPo. TEC_+n_ (*n* = 15, 23, 27, and 84) were formed by adding NTPs (ATP, GTP, and CTP). The resulting complexes were resolved in 5% native PAGE. The gel was scanned by fluorescence imager (TMR channel: left panel shows TMR-labelled σ^E^; Cy5 channel: right panel shows Cy5-labelled DNA). (**I**) Same as panel (D), but for σ^E^ on *sigB* template. Each bar represents a mean of three independent replicates with standard deviations, statistical significance was assigned based on *P*-value <.05 (**** denotes *P *<.001; ns denotes nonsignificance). (**J**) Same as panel (E), but for σ^E^ on *sigB* template. Each bar represents a mean of three independent replicates with standard deviations represented as error bars. Each bar represents a mean of three independent replicates with standard deviations represented as error bars; statistical significance was assigned based on *P*-value <.05 (**, ***, **** denotes *P *<.01, .005, and .001, respectively; ns denotes nonsignificance).

RNAP holoenzyme and RPo were formed as described in the previous section. For pause experiments, two nonradioactive NTPs were added at a final concentration of 125 µM, along with a third radioactive α−P^32^-tagged NTP at 25 µM, and heparin at 25 ng/µl. The mixture was incubated at 37°C for 10 min to form stalled TEC. The absence of the fourth NTP stalled the complex during elongation. To complete transcription (chase), the paused/stalled complexes were supplemented with the fourth NTP at 125 µM, followed by incubation at 37°C for 10 min to generate run-off transcripts. The reaction was halted with formamide dye (80% formamide, 10 mM EDTA, 0.01% Bromophenol Blue, 0.01% Xylene Cyanol), then heated at 95°C for 5 min, and resolved in 15% or 8% (for long templates, >50 nt) denaturing urea–PAGE. Transcripts generated by paused TECs were visualized using the storage phosphor scanner of Amersham Typhoon, GE Healthcare (Figs 2 and 3, and Supplementary Fig. S2).

### Transcription efficiency

The amount of RPo formed during transcription initiation was calculated from the EMSA assay by comparing the fluorescence intensities of the band for free DNA (as the total DNA) and that of the retarded DNA by the complex. The amount of RNA formed by the TEC_+n_ was calculated from the radioactive IVT assay. Thirty-three femtomole of free α-P^32^NTP was loaded onto the gel, which appeared as a dot and its intensity was calculated. The intensity of the RNA formed by TEC_+n_ was compared to that of the free NTP to calculate the amount of transcript formed from a single round of transcription. The amount of calculated transcript directly corresponds to the amount of TEC_+n_. The transcription efficiency values measured as the ratio of the amount of respective paused TECs to that of the RPo (Supplementary Table S7) were calculated as:


\begin{eqnarray*}
{\mathrm{ Amount}}\ \mathrm{ of\ RPo}\ \left( {\mathrm{ {from}\ {EMSA}\ {assay}}} \right) = \frac{{\ (\left[ {\mathrm{ DN}{{\mathrm{ A}}_{{\mathrm{ total}}}}] \times {{i}_{\mathrm{ RPo}}}} \right)}}{{{{i}_0}}}
\end{eqnarray*}

\begin{eqnarray*}
\mathrm{ {Amount}\ of\ {paused}\ TEC} = \frac{{\left( {[x} \right] \times {{i}_{\mathrm{ RNA}}})}}{{\ ({{i}_x}\ \times n\ \times \mathrm{ {reaction}\ {volume}})}}
\end{eqnarray*}

\begin{eqnarray*}
\mathrm{ {Transcription}\ {efficiency}} = \frac{{\mathrm{ {Amount}\ of\ {paused}\ TEC}}}{{\mathrm{ {Amount}\ of\ RPo}}} ,
\end{eqnarray*}

where DNA_total_ is the total amount of DNA, ${{i}_0}$ is the intensity of DNA_total_, and ${{i}_{RPo}}$ is the intensity of DNA bound in RPo from the EMSA assay. The known amount of the free αP^32^-NTP in the dot is ${\mathrm{x}}$ and its intensity from radioactive IVT gel is ${{i}_x}$. The intensity of the band of RNA produced by paused TEC from radioactive IVT gel is denoted as ${{i}_{\mathrm{ RNA}}}$ and the number of αP^32^-NTP in RNA transcript is $n$.

### Fluorescence Anisotropy

First, 5 nM labelled σ and 10 nM unlabelled RNAP core were incubated in 70 µl of transcription buffer (45 mM Tris–HCl, pH 8.0, 5 mM MgCl_2_, 70 mM KCl, 1.5 mM MnCl_2_, 10% glycerol, and 1 mM DTT) for 2 min at 37°C to form holoenzyme. Five nanomolar unlabelled DNA was added and incubated at 37°C for 5 min to form RPo. Three NTPs to form stalled TEC were added at a final concentration of 125 µM, along with 25 ng/µl heparin to ensure single-round transcription, followed by an incubation at 37°C for 10 min. The fluorescence readings were taken using Horiba NanoLog fluorimeter (excitation wavelength = 530 nm, emission wavelength = 580 nm). The fraction of bound complexes (f) was calculated from the formula: fA_0_+ (1 − f)A_1 _= A, where A is the anisotropy value obtained for RPo or TECs, A_0_ is the anisotropy value of free σ, and A_1_ is the anisotropy value of the fully bound σs. The occupancies were corrected for the complexes incompetent to transition to elongation using the formula: E_TEC_ = [E_RPO+NTPs_ − (1 − f)E_RPo_]/f, where f is the transcription efficiency, i.e. the fraction of complexes that successfully transitioned to elongation, and E is the fractional occupancy of σ at RPo or after addition of NTPs. The σ occupancies were plotted against RPo, RPitc, and TECs as bar graphs. Each data set represents a mean of three replicates, and error bars represent the standard deviations. One-way ANOVA was performed for statistical significance testing against a *P*-value of .05 (*, **, ***, **** denotes *P *<.05, .01, .005, and .001, respectively; ns denotes nonsignificance).

### Chromatin immunoprecipitation assay

*Mycobacterium smegmatis* MC^2^155 cells were electroporated with pLAM12 vectors containing *Mtb* σ*^A^*, σ*^F^*, or σ*^E^* genes separately. The colonies were selected for 50 µg/ml kanamycin. A single colony was transferred to 10 ml Middlebrooke 7H9 (MB7H9) broth containing kanamycin (50 µg/ml), 10% Oleic Albumin Dextrose Catalase supplement or OADC, and 0.05% Tween 80. The culture was incubated for 24 h at 37°C with shaking. One millilitres of culture was transferred to 100 ml MB7H9 broth supplied with kanamycin (50 µg/ml), 10% OADC, and 0.05% Tween 80 and incubated at 37°C with shaking till OD_600_ reached 0.2. The cells were induced with 0.02% (low) or 0.2% (high) acetamide and allowed to grow at 37°C with shaking till OD_600_ reached 1.0–1.2. No acetamide was supplied for control (uninduced) cultures. The cells were then crosslinked with 1% formaldehyde for 30 min. Then, 250 mM glycine was used to quench the crosslinking reaction. Cells were centrifuged at 4000 rpm for 10 min at 4°C and washed with ice-cold Tris-Buffered Saline or TBS (20 mM Tris–HCl, pH 7.5, 150 mM NaCl) buffer. The cells were sonicated in lysis buffer (50 mM sodium HEPES, pH 7.5, 150 mM NaCl, 1 mM EDTA, 1% Triton X-100, and 1 mM PMSF). The sample was centrifuged at 13 000 rpm for 15 min. Five microlitres of supernatant was run on 1% agarose gel to visualize the sheared DNA fragments (∼200 bp) after treatment with Proteinase-K. The overexpression of σ factors was validated using western-blot technique using respective antibodies. The sample was precleared using A/G agarose beads and divided into three equal parts, two of which were treated with anti-β and anti-σ antibodies (BioBharati Life Sciences Pvt. Ltd.), respectively. The other part was marked as ‘no antibody control’. All of them were subjected to overnight incubation at 4°C with gentle rocking. Immunoprecipitated (IP) samples were treated with A/G agarose pre-equilibrated by IP dilution buffer (50 mM sodium HEPES, pH 7.5, 150 mM NaCl, 1 mM EDTA, 1% Triton X-100, and Protease Inhibitor Cocktail) and incubated for 3 h at 4°C with rocking. The samples were centrifuged at 2000 rpm for 4 min at 4°C. The beads were sequentially washed with IP wash buffer – I (20 mM Tris–HCl, pH 8.0, 150 mM NaCl, 1 mM EDTA, 1% Triton X-100, and 1 mM PMSF), IP wash buffer – II (20 mM Tris–HCl, pH 8.0, 500 mM NaCl, 1 mM EDTA, 1% Triton X-100, and 1 mM PMSF), IP wash buffer – III (10 mM Tris–HCl, pH 8.0, 1 mM EDTA, 250 mM LiCl, 0.5% NP-40, 0.5% sodium deoxycholate), and Tris-EDTA or TE buffer (10 mM Tris–HCl, pH 7.5, and 1 mM EDTA) with intermittent centrifugation at 2000 rpm for 2 min at 25°C between each wash. The IP samples were eluted out from the beads using 200 µl of elution buffer (25 mM Tris–HCl, pH 7.5, 10 mM EDTA, and 1% sodium dodecyl sulphate) and incubated with Proteinase K (1 µg/µl) at 42°C for 2 h followed by an overnight incubation at 65°C for reverse crosslinking. Purified DNA was eluted using the manufacturer protocol of chromatin immunoprecipitation (ChIP) assay DNA purification kit (Zymo Research). Note that to monitor *M. smegmatis* σ activity without overexpression of *Mtb* σs, 10 times higher amount of cells were used to perform the ChIP assays.

### Quantitative real-time PCR

DNA samples obtained from the ChIP assay were analysed using quantitative PCR (qPCR) method. One-tenth of diluted DNA and specific primer pairs were added to Bio-Rad SYBR Green Supermix (Supplementary Table S8). The PCR reaction was carried out using the Agilent AriaMX real-time PCR system. The obtained C_t_ values were corrected with primer efficiencies (E = 10^−1/slope^) for each primer pair. The primer efficiencies were calculated by plotting C_t_ values against the log of the order of dilution of the DNA used for amplification by the primers. Each specific set of primers was designed to amplify 68–70 bp of DNA spanning across the promoters and downstream coding regions of the respective genes. C_t_ values obtained for β (representing all RNAP on DNA) and σ (representing RNAP holoenzyme on DNA) from qPCR analysis were corrected with ‘no-antibody’ DNA pulldown control C_t_ values (ΔCt_β _= Ct_β_ − Ct_No-Ab_ and ΔCt_σ _= Ct_σ_ − Ct_No-Ab_). The β and σ occupancies were calculated as E ^(−ΔCt)^ where E represents primer efficiency of primers used for the qPCR. Note that E^(−ΔCt)^ values obtained from qPCR using β antibodies representing RNAP were ∼5–20 fold higher compared to the E^(−ΔCt)^ values obtained using σ antibodies (Supplementary Figs S3–S5). Since the ideal ratio of β (RNAP) to σ at the promoter should be 1, the pulldown efficiency of β antibody is likely higher than σ antibody. Therefore, we have normalized the E^(−ΔCt)^ values obtained with both β and σ antibodies with the respective highest E ^(-ΔCt)^ values (observed at the promoter regions). The β and σ occupancies across respective DNA were plotted as bar graphs, and each bar represents a mean of six replicates with standard deviations represented as error bars. One-way ANOVA was performed for statistical significance testing against a *P*-value of .05 (*, **, ***, **** denotes *P *<.05, .01, .005, and .001, respectively; ns denotes nonsignificance).

### Promoter-like motif detection on *Mycobacterial* genes

*Msm* genes were investigated for the occurrence of intragenic promoter-like motifs. In-house biopython script was used for consensus generation and sequence search across template and nontemplate strands of specific genes. For σ^A^-specific genes, the consensus sequences were derived from experimentally validated σ^A^-specific promoter sequences [41–45]. A promoter consensus search was done for −35 (TTGHNH) and −10-like (TANNNT) elements on the *Msm 16S rrnA*, and *sigA* genes. For σ^F^ and σ^E^ specific genes, the promoter motifs (−35 and −10-like) recognized by the respective σ factors were searched across the intragenic regions instead of the consensus sequences, as only a few experimentally validated promoter sequences for these σ factors are known (Supplementary Table S9) [46, 47]. The biopython codes used for sequence search can be accessed at Figshare repository (https://doi.org/10.6084/m9.figshare.30729554).

### RNAP Core-σ FRET experiments

First, 2.5 nM labelled RNAP core derivative was incubated with 5 nM σ in 70 µl transcription buffer (45 mM Tris–HCl, pH 8.0, 5 mM MgCl_2_, 70 mM KCl, 1.5 mM MnCl_2_, 10% glycerol, and 1 mM DTT) for 5 min at 25°C to form holoenzyme. Then, 1 nM of DNA was added with 37°C incubation for 10 min to form RPo. The three NTPs to stall RNAP at nth position on respective DNA were added at a concentration of 125 µM along with 25 ng/µl heparin and incubated at 37°C for 10 min to form stalled elongation complexes. Fluorescence intensities were monitored using Horiba NanoLog fluorimeter. The FRET values of TEC were corrected for the subpopulations that are not competent to form TEC as above. The data obtained were plotted as FRET efficiency, E, calculated as:


\begin{eqnarray*}
F_{\mathrm{ Dem,Dex}}^{{\mathrm{ FRET}}} = \ F_{\mathrm{ Dem,Dex}}^{\mathrm{ DA}} - \frac{{F_{\mathrm{ Dem,Dex}}^\mathrm{ A}*F_{\mathrm{ Aem,Aex}}^{\mathrm{ DA}}}}{{F_{\mathrm{ Aem,Aex}}^\mathrm{ A}}} ,
\end{eqnarray*}

\begin{eqnarray*}
E = \frac{{F_{Dem,Dex}^D - F_{Dem,Dex}^{\textit{FRET}}}}{{F_{Dem,Dex}^D}}
\end{eqnarray*}

where ${\mathrm{F}}_{{\mathrm{Dem}},{\mathrm{Dex}}}^{{\mathrm{FRET}}}$ is attributable to the corrected FRET values (*D* stands for donor; *A* stands for acceptor; and *DA* represents the presence of both donor and acceptor) obtained by donor emission (em) at 520 nm when excited (ex) at 495 nm wavelength and *E* is the efficiency of FRET. Acceptor excitation and emission wavelengths were 550 and 580 nm, respectively.

### Formation of nucleic acid scaffold and TEC

Nucleic acid scaffold was formed by the addition of 25 μM each of template DNA, nontemplate DNA, and RNA in 50 µl annealing buffer (10 mM Tris, pH 8.0, 50 mM NaCl and 1 mM EDTA), incubated at 95°C for 2 min, and gradually cooled (−1°C/minute) to 10°C to form an RNA:DNA hybrid. 50 nM nucleic acid scaffold was incubated with 50 nM Dylight488-labelled RNAP core in 10 µl transcription buffer (45 mM Tris–HCl, pH 8.0, 5 mM MgCl_2_, 70 mM KCl, 1.5 mM MnCl_2_, 10% glycerol, and 1 mM DTT) at 37°C for 10 min to form TEC, eluted through a heparin-sepharose column to remove excess unbound RNAP core and was tested for transcriptional ability using IVT assay; 2.5 nM purified nucleic acid scaffold-bound TECs were added in 70 µl transcription buffer (45 mM Tris–HCl, pH 8.0, 5 mM MgCl_2_, 70 mM KCl, 1.5 mM MnCl_2_, 10% glycerol, and 1 mM DTT) with increasing concentrations of TMR-labelled σ, followed by incubation at 37°C for 5 min, to monitor binding of the respective σ factors to the elongation complex by FRET assay as above.

## Results

### Primary σ factor, σ^A^ undergoes stochastic release during elongation

To monitor the nature of σ^A^ release during early to mature phases of transcription, we used fluorescence-based EMSA with the RPo, RPitc, and TEC stalled at a specific site at the downstream DNA. The RPo was formed using Alexafluor-488 labelled σ^A^ that was preincubated with unlabelled RNAP core before the addition of Cy5 labelled DNA. To stall RNAP at a specific site on DNA (TEC_+n_) we used the promoter DNA fragment in which the first C nucleotide appears at *n* + 1 nucleotide base after the TSS (Fig. 2A). RPitc was formed by the addition of two NTPs to RPo. TEC_+n_ was formed by adding 3 NTPs—ATP, GTP, and UTP to stall the complex at the n^th^ nucleotide due to the absence of CTP. The formation of product RNA transcripts was confirmed by radioactive IVT assay for all TECs (Fig. 2B). For EMSA gels, the fluorescence intensities of σ^A^ and DNA were monitored separately by scanned images of the gel at 488 and Cy5 channels, respectively, in Amersham Typhoon imager (Fig. 2C). The fractional occupancies of σ^A^ for bound DNA were calculated for RPo, RPitc, and TEC from EMSA by estimating the relative amount of σ^A^-488 content in the complexes versus the relative amount of Cy5-labelled DNA representing the total quantity of the complexes (Fig. 2D). The final fractional occupancy of σ^A^ was corrected by the fraction of RPo that were not competent to undergo the transition from transcription initiation to elongation by estimated transcription efficiency values (Fig. 2D). For σ^A^, we used three representative promoter DNA fragments: *sinP3, sigA*, and *rrnAP3*. While *sinP3* is a strong promoter from *B. subtilis* recognized by *Mtb* RNAP-σ^A^ holoenzyme, two *Mtb* specific promoters—*sigA*, the promoter at which σ^A^ positively autoregulates, and *rrnAP3*, the rRNA promoter that acts as a relatively weak promoter in the absence of RbpA and CarD, were used [36, 48].

The fractional occupancies of σ^A^ relative to RPo on *sinP3* promoter were estimated to be ∼95 (±13)%, 77 (±5)%, 72 (±11)%, 63 (±4)% and 49 (±1)% at RPitc, TEC_+16_, TEC_+23_, TEC_+29_, and TEC_+59_ respectively (Fig. 2D). Similar results were obtained with the *sigA* and *rrnAP3* promoters (Supplementary Fig. S6). To validate our findings with a complementary assay that does not involve any separation technique, we performed a fluorescence anisotropy assay to monitor Alexafluor-488 labelled σ^A^ occupancy at TECs (Fig. 2E). The anisotropy values were recorded for free σ^A^, RPo, RPitc, and TEC. As expected, an increase in anisotropy of RPo with respect to free σ was observed due to the binding of RNAP and DNA and represents the anisotropy value of bound σ. Anisotropy assay predicts a decrease in anisotropy value for TEC relative to RPo for σ-release, and an unchanged anisotropy value for σ nonrelease (Fig. 2E). The fraction of bound complexes (f) was calculated from the formula: fA_0_+ (1 − f)A_1 _= A, where A is the anisotropy value obtained for RPo or TECs, A_0_ is the anisotropy value of free σ, and A_1_ is the anisotropy value of the bound σs. The relative occupancies of σ^A^ on the *sinP3* template were 94 (±1)%, 85 (±2)%, 73 (±14)%, 63 (±10)%, and 52 (±10)% at RPitc, TEC_+16_, TEC_+23_, TEC_+29_, and TEC_+59,_ respectively (Fig. 2F). Thus, data from the anisotropy assay corroborate the results from EMSA. The results from both EMSA and anisotropy assays suggest σ^A^ undergoes a gradual decrease in its occupancy at TECs from early to late elongation. This observation corresponds to the existing model for σ^70^ in *E. coli*, which predicts a stochastic release of σ^70^ from the elongating RNAP [8, 9].

### Alternate σ factor σ^F^ is retained on RNAP during elongation *in vitro*

*Mtb* σ^F^ is evolutionarily distant from the principal factor σ^A^ and recognizes significantly different promoter sequences. To test whether σ^F^ follows a distinct pattern of σ-release from σ^A^, we examined the σ^F^ occupancy at RPo and respective TEC_+n_ sites on *usfXP1* and *phoY1* promoters using EMSA and fluorescence anisotropy assay (Fig. 3A and Supplementary Table S4). The formation of product RNA transcripts was confirmed by radioactive IVT assay for all TECs (Fig. 3B). The fractional occupancies of σ^F^ calculated from EMSA at TECs relative to RPo on *usfXP1* template were ∼96 (±5)%, 89 (±5)%, 87 (±2)%, 83 (±2)%, and 82 (±11)% at RPitc, TEC_+17_, TEC_+23_, TEC_+28_, and TEC_+64_, respectively (Fig. 3C and D). The relative occupancies of σ^F^ calculated from the anisotropy assay were 98 (±3)%, 95 (±10)%, 89 (±16)%, 89 (±13)%, and 80 (±11)% at RPitc, TEC_+17_, TEC_+23_, TEC_+28_, and TEC_+64_, respectively (Fig. 3E), which were close to the values obtained from EMSA. Thus, σ^F^ was majorly retained throughout transcription elongation on *usfXP1* template. Similar results were observed for another σ^F^-dependent *phoY1* promoter (Supplementary Fig. S6). Thus, our results reveal that the majority of σ^F^ is retained during elongation in contrast to σ^A^, which is gradually released as the TEC moves along the downstream gene.

### The ECF factor σ^E^ undergoes immediate release from most RNAP during elongation

*Mtb* σ^E^ constitutes σ_2_ and σ_4_ domains to recognize −10 and −35 promoter elements, respectively. We examined the σ^E^ occupancy at RPo and TEC_+n_ for the *sigB* promoter regulated by σ^E^ by stalling RNAP at TEC_+15_, TEC_+23_, TEC_+27_, and TEC_+84_ downstream of the *sigB* promoter (Fig. 3F). The formation of product RNA transcripts was confirmed by radioactive IVT assay for all TECs (Fig. 3G). The fractional occupancies of σ^E^ relative to RPo on *sigB* template were observed as ∼93 (±10)%, 55 (±3)%, 47 (±3)%, 45 (±11)%, and 38 (±3)% at RPitc, TEC_+15_, TEC_+23_, TEC_+27_, and TEC_+84,_ respectively (Fig. 3H and I) using EMSA. The relative occupancies of σ^E^ calculated from anisotropy assay at TECs were calculated as 95 (±5)%, 64 (±12)%, 51 (±4)%, 46 (±13)%, and 44 (±10)% at RPitc, TEC_+15_, TEC_+23_, TEC_+27_, and TEC_+84,_ respectively (Fig. 3J). The results were verified on another σ^E^-specific promoter, *hsp20* by EMSA (Supplementary Fig. S6). Our observations reveal an immediate release of a significant fraction of σ^E^ upon the transition from transcription initiation to elongation.

### *Mtb* σ^F^ exhibits a stable association with TECs, unlike σ^A^ and σ^E^

**Figure 4.**
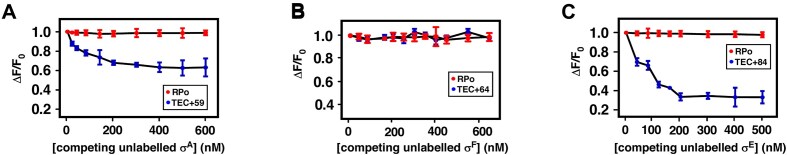
Stabilities of *Mt*b σ factors in RPo and TECs: FRET competition assays.(**A–C**) RPo and TECs were formed with Dylight488-labelled RNAP core and TMR-labelled σ factor (σ^A^, σ^F^, or σ^E^); the complexes were titrated with respective unlabelled σ factors, and the FRET change was monitored with respect to the initial FRET signals (donor quenching was measured at 520 nm with excitation at 495 nm). Each point in the graphs represents the mean of three independent replicates, with standard deviations.

**Figure 5.**
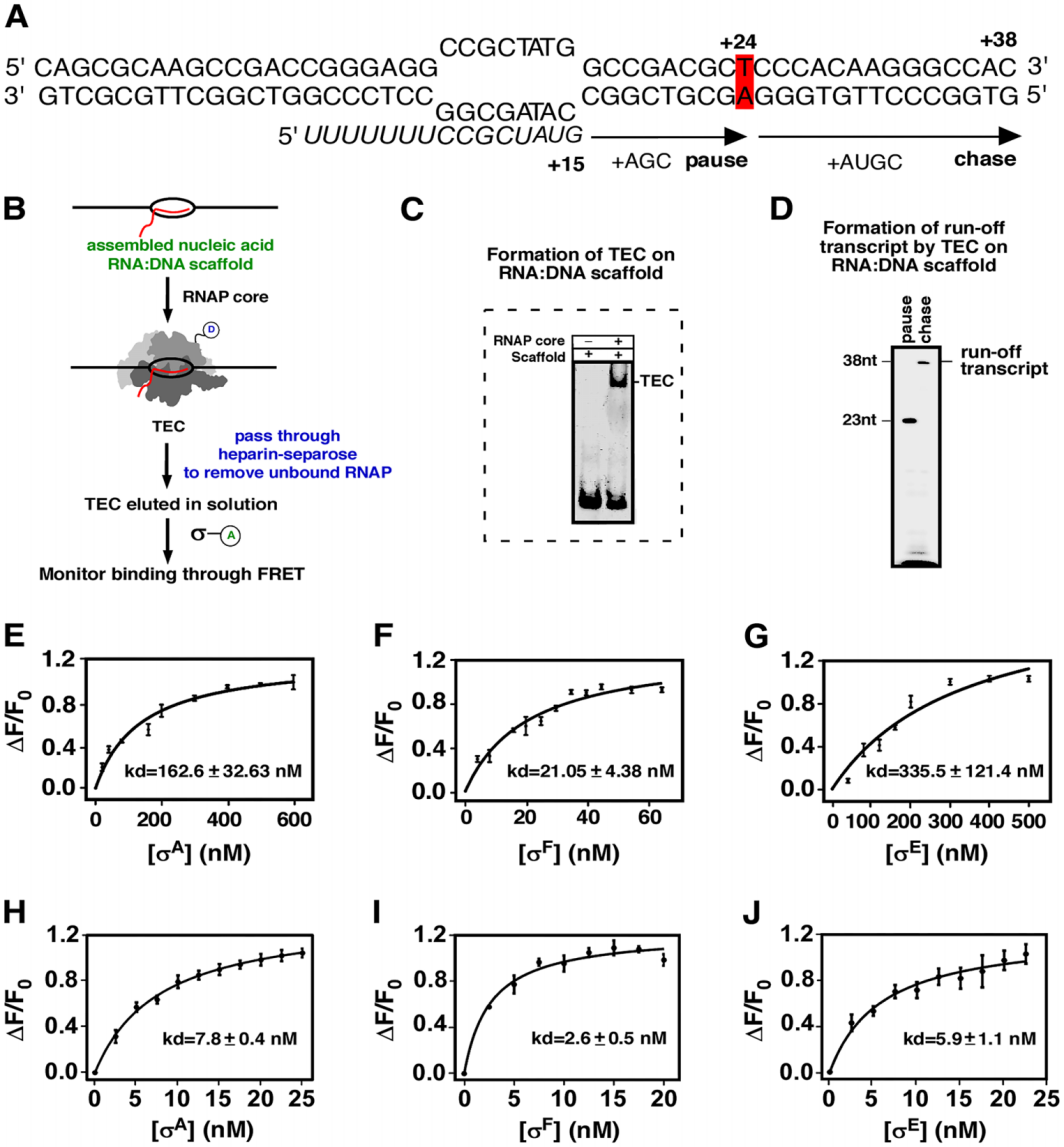
*Mtb* σ binding affinities to TECs formed on RNA:DNA nucleic acid scaffold. (**A**) Sequences of DNA and RNA used to form the nucleic acid scaffold. The RNA (length = 15 nt) extends up to 23 nt in the presence of ATP, GTP, and CTP (pause) and up to 38 nt when additionally supplied with UTP (chase). (**B**) Schematic representation of the RNA:DNA nucleic acid scaffold assembly, followed by formation of TEC by the addition of Dylight488-RNAP core (D denotes donor; ex wavelength = 495 nm and em wavelength = 520 nm). The TEC formed is eluted through the heparin-sepharose column to remove the excess unbound RNAP. Then, 2.5 nM TEC is titrated with increasing concentration of TMR-σ (A denotes acceptor), and the FRET change (donor quenching) is monitored at 520 nm to estimate binding affinities of the respective σ factors to the TEC. (**C**) EMSA assay to monitor the formation of TEC on assembled RNA:DNA nucleic acid scaffold upon the addition of RNAP core. The gel was stained with Sybr Gold. (**D**) IVT assay with the assembled RNA:DNA nucleic acid scaffold. The RNA lengths after pause and chase assays are 23 and 38 nt. (**E–G)** FRET assay to monitor binding of σ factors (σ^A^, σ^F^, and σ^E^) to TEC formed on RNA:DNA nucleic acid scaffold. Each point in the graphs represents the mean of three independent replicates, with standard deviations. (**H–J**) Same as panels (E)–(G), except RNAP core was used instead of TEC. Each point represents a mean of three independent replicates with standard deviations.

To monitor the stability of the σ factor in the σ-containing TECs, we performed FRET competition assays. RPo and TECs were formed using 2.5 nM Dylight-488 labelled RNAP core and 5 nM TMR-σ, which were then challenged by increasing concentrations of competing unlabelled σ. If the stability of the σ factor in the TEC is high, the competing unlabelled σ cannot displace the labelled σ from the TEC. Conversely, if the stability of the σ in the TEC is low, the competing unlabelled σ factor can displace the labelled σ, causing a decline in FRET. As expected, the results showed that all three σ are stably associated with RNAP in RPo, even a 240-fold molar excess of respective unlabelled σ failed to dissociate σ from the complexes (Fig. 4). Interestingly, we observed that as in RPo, σ^F^ was also stably associated with RNAP in TEC. In contrast, 80-fold molar excess of unlabelled σ^A^ and σ^E^ could completely or near-completely dissociate the respective labelled σ from TEC, indicating weak association of these two σ in TEC. The differential stability of three σ factors was also supported by the differential affinity of these σ to RNAP core in TEC. A transcriptionally active TEC was formed by mixing a fluorescently labelled RNAP core with DNA-RNA hybrid scaffold (Fig. 5A–D). The dissociation constants (K_d_) for σ binding to TEC were estimated to be 21.05 ± 4.38 nM for σ^F^, 162.0 ± 32.6 nM for σ^A^, and 335.0 ± 121.4 nM for σ^E^ (Fig. 5E–G). In comparison, the estimated dissociation constants of binding of these σ to RNAP core are 2.6 ± 0.5 nM for σ^F^, 7.8 ± 0.4 nM for σ^A^, and 5.9 ± 1.1 nM for σ^E^ (Fig. 5H–J). Thus, all three σ exhibit similar and high affinity to RNAP core alone. The affinity to RNAP in TEC was greatly reduced for σ^E^, moderately reduced for σ^A^, whereas the affinity of σ^F^ is reduced, but remained significantly higher compared to the other two σs. Thus, our data explain the release of σ^A^ and σ^E^ due to their significantly reduced affinities to RNAP upon formation of the elongation complex, whereas σ^F^ remained bound to TEC as it retains significantly higher affinity to the elongating RNAP. The stability of σ^F^ in TEC is comparable to its stability in RPo. Thus, the observed retention of σ^F^ in TEC is not due to the rebinding of the σ to the TEC.

### Elongation factor NusA promotes the release of σ^A^ from TECs, while initiation factors, RbpA and CarD and initial transcribing sequence has no effect on σ release

**Figure 6.**
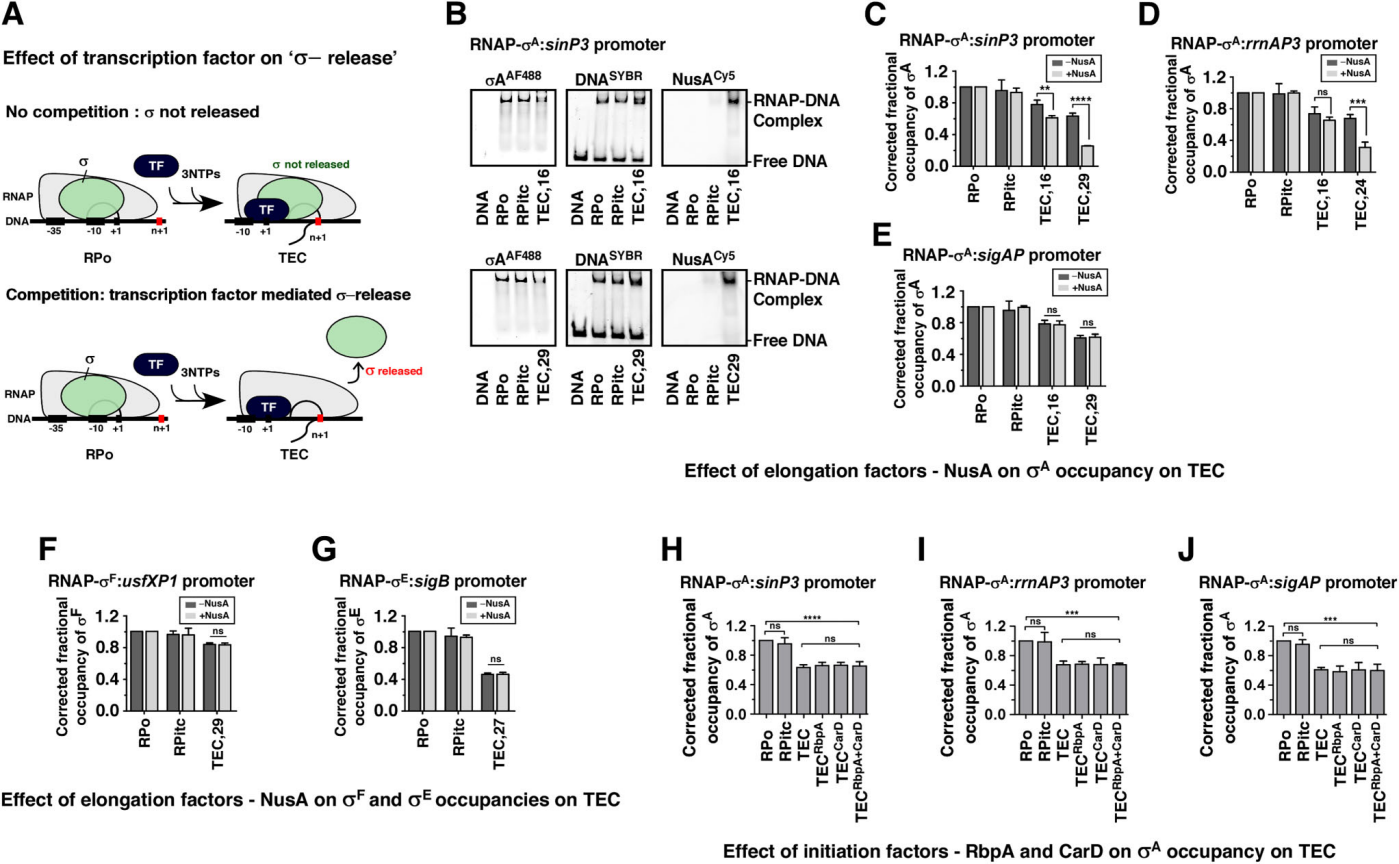
Effect of *Mtb* transcription factors (TFs)—NusA, RbpA, and CarD—on σ-release from TECs: EMSA assay. (**A**) Schematic illustration of TF mediated competitive σ nonrelease/release from TEC. (**B**) EMSA assay: Effect of NusA on σ^A^ release monitored on RPo, RPitc, and TECs at *sinP3* template (*n* = 16 and 29) using EMSA. (**C**) Bar graph comparing σ^A^ occupancies at TECs in the absence (dark grey) and presence (light grey) of NusA on *sinP3* template. Each bar represents a mean of three independent replicates with standard deviations represented as error bars; statistical significance was assigned based on *P*-value <.05 (**, *** denotes *P *<.01, and .005, respectively). (**D, E**) Same as panel (C), except on *rrnAP3* and *sigA* template, respectively. Each bar represents a mean of three independent replicates with standard deviations represented as error bars; statistical significance was assigned based on *P*-value <.05 (*** denotes *P *<.005; ns denotes nonsignificance). (**F, G**) Bar graph comparing σ^F^ and σ^E^ occupancies at TECs in the absence (dark grey) and presence (light grey) of NusA on *usfXP1* and *sigB* template, respectively. Each bar represents a mean of three independent replicates with standard deviations represented as error bars; statistical significance was assigned based on *P*-value <.05 (ns denotes nonsignificance). (**H–J**) Bar graph comparing σ^A^ occupancies at TECs in presence of RbpA, CarD, and RbpA + CarD on *sinP3, rrnAP3*, and *sigA* template. Each bar represents a mean of three independent replicates with standard deviations represented as error bars; statistical significance was assigned based on *P*-value <.05 (***, **** denotes *P *< .005 and .001, respectively; ns denotes nonsignificance).

Earlier reports suggest that NusA, an essential elongation factor, competitively displaces the weakly bound σ^70^ from TECs during elongation [6]. It functionally destabilizes RNA hairpin structures that can impede RNAP during elongation [49]. NusA enhances transcriptional readthrough of untranslated RNA in the presence of *nut* sites (*boxB, boxA*, and *boxC*) at the *rrnA* gene [50]. However, recent findings demonstrate that NusA interaction with paused TECs is sequence nonspecific and does not involve a consensus [51]. NusA-specific *nut* sites are present on *Mtb rrnAP3* template DNA immediately downstream of the transcriptional start site, but not present on *sinP3* and *sigA* template DNA [51]. To test the effect of *Mtb* NusA on σ-release, we monitored σ^A^ occupancy in the presence of NusA for all three promoters—*sinP3, rrnAP3*, and *sigA*—using EMSA (Fig. 6 and Supplementary Fig. S7). In the presence of NusA, the fractional σ^A^ occupancies on RNAP for *sinP3* template DNA were observed to be ∼93 (±6)%, 61 (±3)%, and 25 (±1)% at RPitc, TEC_+16_, and TEC_+29_, respectively, compared to the fractional σ^A^ occupancies of 95 (±13)%, 77 (±5)%, 63 (±4)% at RPitc, TEC_+16_, and TEC_+29_, respectively, in the absence of NusA (Fig. 6A–C). We further observed that NusA is able to bind TEC (Fig. 6B and Supplementary Fig. S7). The result shows enhancement of σ^A^ release from the TEC on *sinP3* DNA template in the presence of NusA (Fig. 6C). Similar results were obtained for the *rrnAP3* template, suggesting NusA enhances σ^A^ release in both the DNA (Fig. 6D and Supplementary Fig. S7). However, no effect of NusA on σ^A^ occupancy at TEC was observed for *sigA* template (Fig. 6E and Supplementary Fig. S7). Thus, a NusA-σ^A^ cycle exists on specific genes (*sinP3* and *rrnAP3* DNA template), where NusA enhances σ^A^ release from RNAP during elongation. We did not observe any effect of NusA on σ^F^ release from TEC (Fig. 6F and Supplementary Fig. S8). The addition of NusA did not enhance σ^E^ release from TECs (Fig. 6G and Supplementary Fig. S8).

Since RbpA and CarD are known to promote transcription initiation in σ^A^-dependent promoters [34, 36, 52, 53] we have separately monitored the effect of these TFs on σ-release. We observed that, although RbpA and CarD bind to both RPo and TEC, these factors do not influence the σ^A^-release (Fig. 6H–J and Supplementary Fig. S9).

To examine whether the initial transcribing sequence (ITS) influences σ-release, we performed EMSA-based assays in which the ITS region of a given promoter DNA template was replaced with that from another gene. Several promoter DNA constructs were generated to evaluate the σ-release behaviour of all three σ factors. The results showed that substitution of the ITS led to the synthesis of distinct abortive transcripts, indicating altered early transcription dynamics; however, the overall pattern of σ-release from the TEC remained unchanged (Supplementary Figs S10–S12).

### *Mtb* σ factors are differentially retained throughout transcription elongation *in vivo*

**Figure 7.**
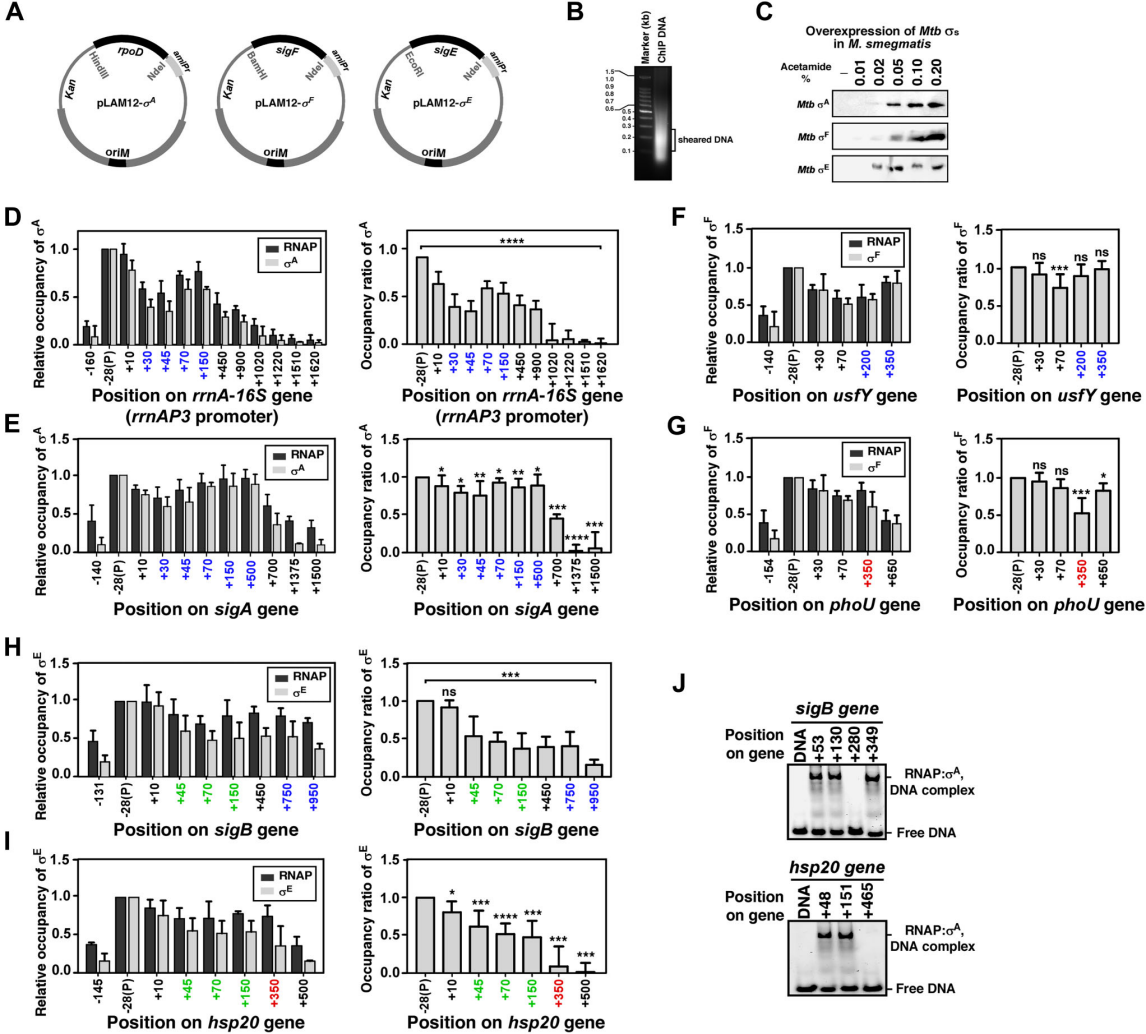
Relative occupancies of *Mtb* σ factors at genes of *Msm in vivo*: ChIP assays. (**A**) pLAM12 plasmids used to overexpress *Mtb* σ factors in *M. smegmatis*. (**B**) Sheared chromatin run on 1% agarose gel after sonication. (**C**) *Mtb* σ factors expressed in *M. smegmatis* upon acetamide induction. Cells were crosslinked and lysed by sonication. The lysate was resolved using 10% SDS–PAGE, and σ factors were visualized using respective antibodies by western blot. (**D**)** Left**: Occupancies of σ^A^ (representing RNAP holoenzyme) and β (representing total RNAP) at the promoter (−28 to +10) and coding region (+30 to +1620) of *rrnA* gene. The positions on the gene denote the mid-point of the DNA fragments (∼60–70 bp) amplified by real-time PCR; positions marked blue, encompass cognate σ binding sites; red, noncognate σ binding sites; green, both cognate and noncognate σ binding sites. **Right**: Occupancy ratio of σ^A^ to RNAP at specific positions on the downstream gene after the promoter upstream occupancy at the control site was subtracted. The standard deviations are represented as error bars; each bar represents a mean of six independent replicates; statistical significance was assigned based on *P*-value <.05 (*, **, ***, **** denotes *P *<.05, .01, .005, and .001, respectively; ns denotes nonsignificance). (**E**) As panel (D), but on *sigA* gene at promoter (−28 to +10) and coding region (+30 to +1500). (**F**) As panel (D), except for σ^F^ on *usfY* gene at promoter (−28) and coding region (+30 to +350). **G**, Same as D, except for σ^F^ at promoter (−28) and coding region (+30 to +650) of *phoU* gene. (**H**) As panel (D), except for σ^E^ at promoter (−28 to +10) and coding region (+45 to +950) of *sigB* gene. (**I**) As panel (D), but for σ^E^ at promoter (−28 to +10) and coding region (+45 to + 500) of *hsp20* gene. (**J**)** Upper**: Nonpromoter DNA fragments (downstream regions) of *Msm sigB* gene with σ^A^ binding consensus at positions +53, +130, and +349 (as mid-points of the intragenic fragments) post-TSS and lacking the consensus (at position +280, post-TSS) were incubated with RNAP-σ^A^ holoenzyme. The reaction was resolved using 5% native PAGE. The gel was stained with SYBR gold dye and scanned using 488-channel of Amersham Typhoon imager. DNA-bound complexes can be seen as retarded bands. **Lower**: Nonpromoter fragments of *Msm hsp20* gene were checked for RNAP-σ^A^ holoenzyme binding at positions with (+48 and +151, post-TSS) and without (at +465, post-TSS) a σ^A^ binding consensus.

To further investigate the nature of σ release inside bacteria, we examined the RNAP:σ distribution of σ^A^, σ^F^ and σ^E^  *in vivo* at their respective promoters and downstream coding regions spanning along the genes using ChIP assay followed by real-time or qPCR [9]. Respective *Mtb* σ factors were overexpressed in *M. smegmatis* (*Msm*) cells to form a holoenzyme with RNAP core (Fig. 7A–C). Earlier studies on the ability of *Mtb* σs to function with *Msm* RNAP [54] were confirmed by our IVT assays (Supplementary Fig. S1I-K). Cells were crosslinked and lysed before the RNAP-pulldown was performed using β and σ antibodies separately. The β and σ occupancies across respective promoter DNA and downstream genes were determined by the Ct values from qPCR using the pulled-down DNA and were plotted as bar graphs (Fig. 7D–I).

The σ^A^-specific DNA used for ChIP/qPCR assays were *Msm* homologues of *sigA* and 16S rRNA genes, respectively, having near identical promoter sequences of *sigA* and *rrnAP3* promoter templates used in the *in vitro* assays. Similarly, relative occupancies of σ^F^ and RNAP were determined for *usfY* and *phoU* genes (for respective *Msm* homologues *of Mtb* genes *usfX* and *phoY*), and of σ^E^ were determined for *Msm* homologues of *sigB* and *hsp20*. For each of the ChIP assays, as a control, we monitored the relative σ occupancy at a site upstream of the promoter. Intriguingly, a significant σ occupancy was observed for each of the control sites for each σ.

We observed a gradual decrease in σ^A^ occupancies along the downstream gene of the 16S rRNA except at positions +70 and +150 (Fig. 7D). Notably, the *rrnA* gene encompasses a *nut* site at the initial stretch (2–63 bp). Thus, NusA possibly facilitates σ^A^ release. The *in vivo* observations are consistent with our *in vitro* results that indicated a stochastic σ release at the *rrnA* gene, further facilitated by the presence of NusA. The elevated levels of σ^A^ at certain sites of the downstream gene are possibly due to the prevalence of frequently occurring σ^A−^specific promoter-like consensus motifs that are expected to facilitate σ^A^-RNAP binding (sites that encompass cognate σ binding sites were marked in blue in Fig. 7D). Consequently, we verified using EMSA assay that the DNA fragments encompassing the high σ-occupancy region is able to bind the σ-RNAP holoenzyme (Fig. 7J). An increase in σ occupancy in the coding regions may be a result of holoenzyme binding from the bulk solution to the intragenic promoter-like sequences, leading to elevated CHIP signals at these sites. A decrease in both RNAP and σ occupancies after > ∼+1300 position possibly reflects termination events if promoter-like sequences are not present to stall the RNAP or to facilitate trans-mediated binding of the holoenzyme. The *sigA* gene of *Msm*, which lacks NusA binding consensus, showed significantly higher amounts of σ^A^ occupancies throughout the length of the gene (Fig. 7E), possibly due to the prevalence of σ^A^ binding sites at the regions investigated (Supplementary Table S9).

The occupancies of σ^F^ for RNAP on both *usfY* and *phoU* genes were high and close to the occupancy of σ^F^ at the promoter regions throughout the genes (Fig. 7F–G). Only at position +350 of *phoU*, the relative occupancy is relatively lower than the other positions (Fig. 7G). We observed that this intragenic region contains an σ^A^-specific consensus motif (sites that encompass noncognate σ binding sites were marked in red in Fig. 7G). Thus, the binding of RNAP-σ^A^ holoenzyme at this position may affect the estimated ratio of RNAP core and σ^F^. We also observed a few σ^F^ binding sites present on the downstream sequences, possibly enhancing σ^F^-mediated transcriptional pausing events during transcription elongation (e.g. at position +350 of *usfY* gene). Nevertheless, the *in vivo* results support the *in vitro* results that the majority of σ^F^ is retained with the TEC. σ^E^-specific genes *sigB* and *hsp20* showed a gradual release of σ^E^  *in vivo* (Fig. 7H and I). A decrease in both RNAP and σ occupancies after > ∼+500 position at the *hsp20* gene possibly reflects termination events. Both the *sigB* and *hsp20* genes contain putative σ^A^-binding sites at the downstream regions. EMSA with representative DNA fragments containing σ^A^-binding sites (Supplementary Table S10) confirmed the binding of RNAP-σ^A^ holo to these DNA regions (Fig. 7J). This also corresponds to the elevated levels of RNAP occupancy at certain regions observed using CHIP. Both *in vitro* and *in vivo* results with σ^E^ indicate a trend of σ^E^ release; however, the *in vitro* result shows the immediate release of ∼50%–60% σ^E^, whereas the release observed *in vivo* is found to be more gradual than immediate.

All *in vivo* ChIP assays were conducted by expressing the respective σ factors in *M. smegmatis*. To rule out the possibility that σ overexpression influenced the ChIP signals, we performed control assays under conditions of no σ expression and low-level σ expression (Supplementary Figs S3–S5). In the absence of σ expression, the ChIP signals showed negligible enrichment of σ^F^, σ^E^ and RNAP in both promoter and coding regions (Supplementary Figs S4 and *S*5), indicating that transcription from these promoters remains inactive without σ.

Under low-level expression of σ^F^ and σ^E^, the ChIP signals were ∼10-fold less compared to those obtained under higher σ expression but exhibited similar overall patterns of σ occupancies (Supplementary Figs S4 and S5), consistent with the trends shown in Fig. 7F–I.

In contrast, significant σ^A^ occupancies were detected even without its overexpression (Supplementary Fig. S3). This pattern reflects the endogenous *in vivo* distribution of *M. smegmatis* σ^A^ across the two genes analysed. The distribution of *M. smegmatis* σ^A^ relative to the RNAP core within coding regions closely mirrors that of *M. tuberculosis* σ^A^, suggesting a gradual release of σ during transcription elongation. This observation is consistent with the observed *in vitro* stochastic release of Mtb σ^A^. The observed basal level *M. smegmatis* σ^A^ occupancies in the genes indicate that the ChIP signals from both *Mtb* σ^A^ and *M. smegmatis* σ^A^ contribute to the observed σ^A^ occupancies in Fig. 7D and E.

The data obtained for all RNAP:σ occupancy showed a general trend of high RNAP occupancy throughout the entire length of the genes in *M. smegmatis* due to the abundance of σ-binding sites within the intragenic region. Fresh binding of holoenzyme from solution and σ-induced pausing by the translocating holoenzyme that produced the higher CHIP signal cannot be distinguished in these assays. Nevertheless, the *in vivo* results indicate a differential mode of σ retention/release for three different σ factors.

### The σ domains, σ_3_ and σ_2_, maintain stable association with RNAP at TEC and facilitate σ^F^ retention

**Figure 8.**
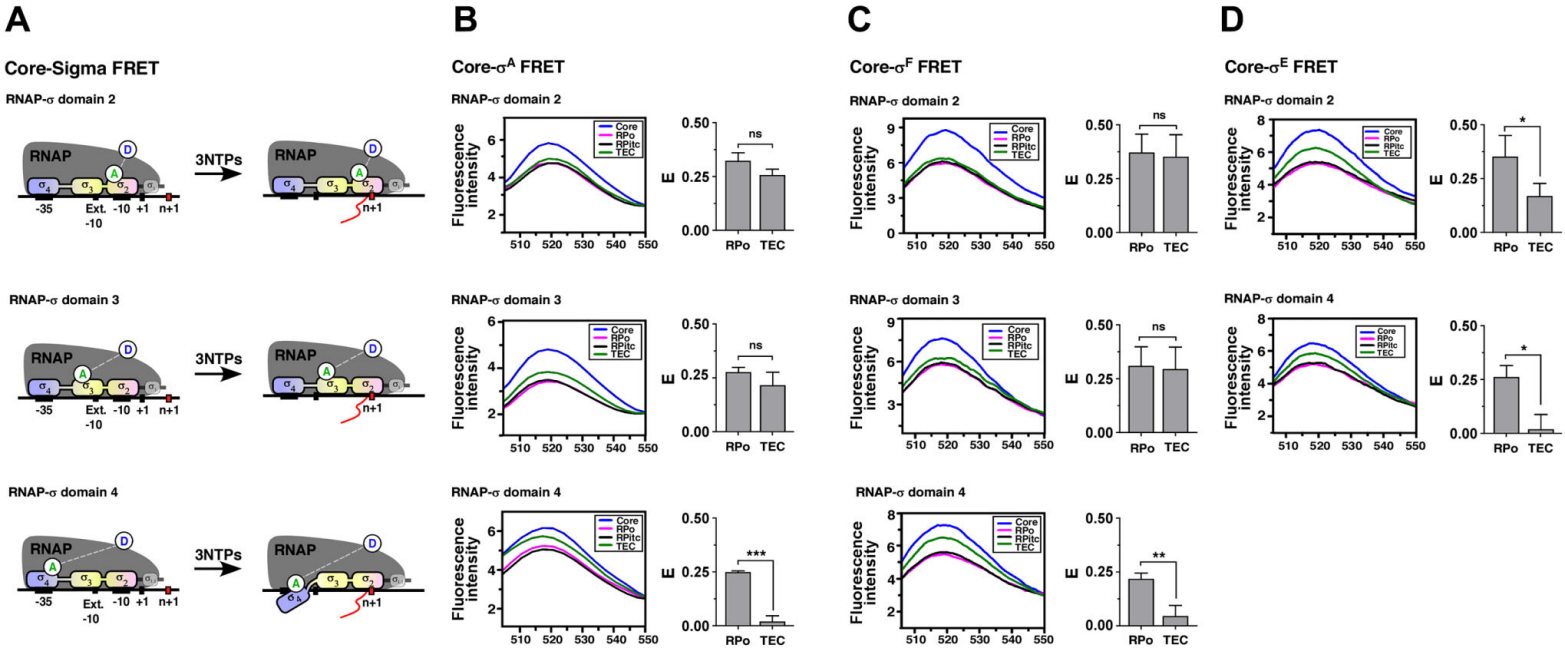
Retention or dissociation/rearrangement of different σ domains with respect to RNAP core at RPo and TEC: FRET assays. (**A**) Schematic representation of biomolecular FRET between RNAP core and σ domains. RNAP core labelled with Dylight-488 and σ domain 2, 3, or 4 labelled with TMR (acceptor) as FRET pairs. (**B**)** Left**: Donor emission spectrum of RNAP core, RPo, RPitc, and TEC. Dylight488-RNAP core and TMR-σ^A^ (labelled at residue positions 331, 400, and 472 for region 2, 3, and 4, respectively) were incubated with *sinP3* template to form RPo. Stalled TEC at +29 was formed and FRET signals were monitored (at excitation: 495 nm, emission: 520 nm). **Right**: Corrected FRET efficiency values (denoted as E) were calculated for RPo and TEC and plotted as bar graphs. Each bar represents a mean of three independent replicates with standard deviations; statistical significance was assigned based on *P*-value <.05 (*, **, *** denotes *P *< .05, .01, and .005, respectively; ns denotes nonsignificance). (**C**) As panel (B), except σ^F^, labelled with TMR at region 2, 3, and 4 (at respective residue positions 47, 156, and 235), were used to form RPo and TEC on *usfXP1* template (*n* = 28). FRET signals were monitored (at excitation: 495 nm, emission: 520 nm), and efficiency values were plotted against RPo and TEC. (**D**) As panel (B), except σ^E^, labelled with TMR at region 2, and 4, (at respective residue positions 92 and 204), were used to form RPo and TEC on *sigB* template (*n* = 27). FRET signals were monitored (at excitation: 495 nm, emission: 520 nm), and efficiency values were plotted against RPo and TEC.

We performed FRET assays to monitor the interaction of different σ domains with RNAP in the context of RPo and TEC formed with RNAP core labelled with a donor fluorophore (Dylight 488-phosphine) at the 359th residue of β’ subunit using unnatural amino acid incorporation (Fig. 8A–D and Supplementary Fig. S1A–C). Individual σ regions of σ^F^ were labelled with acceptor fluorophore TMR-maleimide at a single cysteine residue, natural or artificially incorporated at the 47th residue of σ_2_, 156th residue of σ_3_, and 235th residue of σ_4_ domain. RPo and TEC were formed with labelled RNAP core, labelled σ^F^ and unlabelled *usfXP1* DNA fragment as described before. The donor quenching was monitored to estimate the FRET between RNAP core and σ^F^ domains in RPo and TEC. FRET values of TEC were finally corrected for the subpopulations that are not competent to form TEC.

No significant FRET change was observed for RNAP-σ_2_ and RNAP-σ_3_ in the context of RPo and TEC_+28_, indicating a continued association of σ_2_ and σ_3_ with RNAP at RPo to TEC (Fig. 8C). In contrast, the FRET efficiency between RNAP and σ_4_ decreased markedly from RPo to TEC, indicating displacement or structural rearrangement of this region relative to RNAP in the TEC (Fig. 8C). These observations suggest that σ^F^ remains associated with RNAP in the TEC through the stable interactions of σ_2_ and σ_3_, despite less stable or loss of interaction of σ_4_ with RNAP.

Similar FRET assays were performed to examine the spatial organization of individual regions of σ^A^ and σ^E^ in RPo and in TEC_+29_ or TEC_+27_, respectively (Fig. 8B and D). The occupancies of σ^A^ and σ^E^ in TEC_+29_ and TEC_+27_ were ∼73% and 46%, respectively (Figs 2F and 3J). For σ^A^ molecules that remained bound in the TEC, σ_2_ and σ_3_ showed minimal rearrangement relative to RNAP, whereas σ_4_ underwent a pronounced conformational change compared to its position in RPo.

In the case of σ^E^, FRET between σ_2_ and the RNAP core decreased substantially in TEC relative to RPo, indicating partial rearrangement of this domain in TEC (Fig. 8D). Furthermore, σ^E^ region 4 exhibited a higher change in FRET signal, indicating that the σ_4_ of σ^E^, which remained bound to TEC, undergoes significant rearrangement compared to RPo.

## Discussion

Here, we have observed differential release or retention of three different σ factors of *Mtb* upon the transition from transcription initiation to elongation: σ^A^ and σ^E^ are stochastically released from the TEC, with σ^E^ is released almost immediately after formation of 15 nt RNA, whereas most of the σ^F^ are retained. Earlier studies have shown that *Eco* σ^70^, having all four conserved structural domains σ_1.1_, σ_2_, σ_3_, and σ_4_, is stochastically released from the elongating RNAP [8, 9]. Thus, it is not surprising that σ^A^, having a similar structural organization as σ^70^, shows a similar nature of σ release as σ^70^ during transcription elongation. The presence of NusA enhances the extent of σ release from certain TEC with σ^A^, both in the presence and absence of NusA binding sites, but not from all TEC with σ^A^ and does not have any effect on σ release from TEC with σ^E^ and σ^F^. Transcription initiation factors, CarD and RbpA of *M. tuberculosis*, do not influence σ release or retention in TEC for any of these three σ factors.

Mechanistically, the extent of σ association with RNAP during elongation correlates with their structural organization. *Mycobacterium tuberculosis* σ^A^, like *E. coli* σ^70^, contains all four conserved domains (σ_1.1_–σ_4_) and undergoes stochastic release upon formation of the elongation complex, likely due to displacement of σ_4_ by the nascent RNA and σ_3_/σ_4_ linker rearrangement. In contrast, σ^E^, lacking σ_1.1_ and σ_3_, shows an immediate loss of interaction with RNAP upon elongation. The absence of σ_3_—critical for contacts with the RNAP subunits—reduces its overall affinity for RNAP, and the extended σ_2_/σ_4_ linker that traverses the RNA exit channel introduces steric conflicts with the emerging RNA, promoting σ release [55].

σ^F^, however, is retained with the elongating RNAP despite the rearrangement of σ_4_. FRET analyses revealed that σ^F^ regions 2 and 3 remain stably engaged with RNAP in the TEC, providing sufficient anchorage for its retention. Notably, the σ_1.1_ domain interacts with all three σ_2_, σ_3_, and σ_4_ domains when not bound to the RNAP core (free state), resulting in the formation of a compact state [56–58]. These interactions are missing when σ is in a bound state with the RNAP core [56–60]. The interactions of σ_1.1_ with the other σ domains in the free state are mutually incompatible with the bound state and could compete for the binding of σ to RNAP and destabilize the interaction of σ to RNAP. The absence of σ_1.1_, which in σ^A^ destabilizes holoenzyme interactions through intramolecular domain competition, likely contributes to σ^F^ retention. The role of σ_1.1_ in its release or retention in TEC is also evident in the recent study [25]. *Bacillus subtilis* σ^A^ which contains a relatively smaller σ_1.1_ and a different architecture than *E. coli* σ^70^_1.1_ is retained in TEC, while *E. coli* σ^70^ undergoes stochastic release upon formation of TEC. Removal of region 1.1 renders σ^70^ stably retained in TEC. Thus, σ^F^ may represent an adaptation where loss of σ_1.1_ eliminates an intrinsic trigger for release, resulting in a holoenzyme competent for sustained elongation. Based on our observation and a previous study [25], we propose that the σ factors that contain all the σ domains, except σ_1.1,_ will be retained in TEC. This differential σ retention in TEC points to an evolved regulatory mechanism that fine-tunes transcriptional dynamics and stress adaptation in mycobacteria.

Physiologically, these varying σ dynamics may underlie diverse transcriptional strategies during growth and stress. σ^A^, the housekeeping factor, must recycle rapidly to support high transcriptional turnover, consistent with its stochastic release and NusA-facilitated dissociation. σ^E^, an ECF factor activated under envelope stress, shows rapid release from RNAP, likely allowing swift reallocation of RNAP core to alternative σs and possible rapid reprogramming of the transcriptome during stress transitions. In contrast, σ^F^—primarily active under stationary phase and stress conditions—remains tightly bound to RNAP, possibly ensuring transcriptional continuity of genes essential for persistence. The prolonged association of σ^F^ with elongating RNAP could also enhance transcriptional pausing or facilitate recruitment of antitermination factors, thereby stabilizing expression of long operons or stress-protective genes.

Our data therefore suggest that σ release is not a universal or obligatory step in transcription but rather a σ-specific, mechanistically governed process that modulates transcriptional flexibility. The retention of σ^F^ likely maintains a subset of transcriptional programs independent of σ exchange, while σ^A^ and σ^E^ promote dynamic transcriptome reshaping through σ turnover. This nuanced control of σ retention and release may be a critical component of the mycobacterial transcriptional strategy that supports adaptation to changing environments and contributes to persistence within host macrophages.

## Supplementary Material

gkaf1459_Supplemental_File

## Data Availability

The data underlying this article are available in the article and the online supplemental material. The biopython codes used in the study are available online at Github repository https://github.com/biopython-jcbose/sigma_cycle_project.git and on Figshare at https://doi.org/10.6084/m9.figshare.30729554.

## References

[1] Ishihama A . Functional modulation of *Escherichia coli* RNA polymerase. Annu Rev Microbiol. 2000;54:499–518. 10.1146/annurev.micro.54.1.499. 11018136

[2] Jishage M, Iwata A, Ueda S. et al. Regulation of RNA polymerase sigma subunit synthesis in *Escherichia coli*: intracellular levels of four species of sigma subunit under various growth conditions. J Bacteriol. 1996;178:5447–51. 10.1128/jb.178.18.5447-5451.1996. 8808934 PMC178365

[3] Piper SE, Mitchell JE, Lee DJ. et al. A global view of *Escherichia coli* Rsd protein and its interactions. Mol Biosyst. 2009;5:1943–7. 10.1039/b904955j. 19763331

[4] Burgess RR, Travers AA. *Escherichia coli* RNA polymerase: purification, subunit structure, and factor requirements. Fed Proc. 1970;29:1164–9. 4910267

[5] Burgess RR, Travers AA, Dunn JJ. et al. Factor stimulating transcription by RNA polymerase. Nature. 1969;221:43–6. 10.1038/221043a0. 4882047

[6] Gill SC, Weitzel SE, von Hippel PH. *Escherichia coli* sigma 70 and NusA proteins. I. Binding interactions with core RNA polymerase in solution and within the transcription complex. J Mol Biol. 1991;220:307–24. 10.1016/0022-2836(91)90015-X. 1856861

[7] Metzger W, Schickor P, Meier T. et al. Nucleation of RNA chain formation by *Escherichia coli* DNA-dependent RNA polymerase. J Mol Biol. 1993;232:35–49. 10.1006/jmbi.1993.1368. 7687297

[8] Shimamoto N, Kamigochi T, Utiyama H. Release of the sigma subunit of *Escherichia coli* DNA-dependent RNA polymerase depends mainly on time elapsed after the start of initiation, not on length of product RNA. J Biol Chem. 1986;261:11859–65. 10.1016/S0021-9258(18)67321-1. 2427513

[9] Raffaelle M, Kanin EI, Vogt J. et al. Holoenzyme switching and stochastic release of sigma factors from RNA polymerase *in vivo*. Mol Cell. 2005;20:357–66. 10.1016/j.molcel.2005.10.011. 16285918

[10] Lonetto M, Gribskov M, Gross CA. The sigma 70 family: sequence conservation and evolutionary relationships. J Bacteriol. 1992;174:3843–9. 10.1128/jb.174.12.3843-3849.1992. 1597408 PMC206090

[11] Gribskov M, Burgess RR. Sigma factors from *E. coli, B. subtilis*, phage SP01, and phage T4 are homologous proteins. Nucleic Acids Res. 1986;14:6745–63. 10.1093/nar/14.16.6745. 3092189 PMC311678

[12] Gruber TM, Gross CA. Multiple sigma subunits and the partitioning of bacterial transcription space. Annu Rev Microbiol. 2003;57:441–66. 10.1146/annurev.micro.57.030502.090913. 14527287

[13] Helmann JD, Chamberlin MJ. Structure and function of bacterial sigma factors. Annu Rev Biochem. 1988;57:839–72. 10.1146/annurev.bi.57.070188.004203. 3052291

[14] Feklístov A, Sharon BD, Darst SA. et al. Bacterial sigma factors: a historical, structural, and genomic perspective. Annu Rev Microbiol. 2014;68:357–76. 10.1146/annurev-micro-092412-155737. 25002089

[15] Hansen UM, McClure WR. Role of the sigma subunit of *Escherichia coli* RNA polymerase in initiation. II. Release of sigma from ternary complexes. J Biol Chem. 1980;255:9564–70. 10.1016/S0021-9258(18)43429-1. 7000759

[16] Kang JY, Olinares PDB, Chen J. et al. Structural basis of transcription arrest by coliphage HK022 Nun in an *Escherichia coli* RNA polymerase elongation complex. eLife. 2017;6:e25478 10.7554/eLife.25478. 28318486 PMC5386594

[17] Krummel B, Chamberlin MJ. RNA chain initiation by *Escherichia coli* RNA polymerase. Structural transitions of the enzyme in early ternary complexes. Biochemistry. 1989;28:7829–42. 10.1021/bi00445a045. 2482070

[18] Vassylyev DG, Vassylyeva MN, Perederina A. et al. Structural basis for transcription elongation by bacterial RNA polymerase. Nature. 2007;448:157–62. 10.1038/nature05932. 17581590

[19] Sengupta S, Prajapati RK, Mukhopadhyay J. Promoter escape with bacterial two-component σ factor suggests retention of σ region two in the elongation complex. J Biol Chem. 2015;290:28575–83. 10.1074/jbc.M115.666008. 26400263 PMC4653711

[20] Bar-Nahum G, Nudler E. Isolation and characterization of sigma(70)-retaining transcription elongation complexes from *Escherichia coli*. Cell. 2001;106:443–51. 10.1016/S0092-8674(01)00461-5. 11525730

[21] Mukhopadhyay J, Kapanidis AN, Mekler V. et al. Translocation of σ70 with RNA polymerase during transcription: fluorescence resonance energy transfer assay for movement relative to DNA. Cell. 2001;106:453–63. 10.1016/S0092-8674(01)00464-0. 11525731

[22] Harden TT, Wells CD, Friedman LJ. et al. Bacterial RNA polymerase can retain σ70 throughout transcription. Proc Natl Acad Sci USA. 2016;113:602–7. 10.1073/pnas.1513899113. 26733675 PMC4725480

[23] Yang X, Molimau S, Doherty GP. et al. The structure of bacterial RNA polymerase in complex with the essential transcription elongation factor NusA. EMBO Rep. 2009;10:997–1002. 10.1038/embor.2009.155. 19680289 PMC2750059

[24] Yarnell WS, Roberts JW. The phage lambda gene Q transcription antiterminator binds DNA in the late gene promoter as it modifies RNA polymerase. Cell. 1992;69:1181–9. 10.1016/0092-8674(92)90639-T. 1535556

[25] Tewary A, Sengupta S, Mukherjee S. et al. *Bacillus subtilis* σ(A) and *Escherichia coli* σ(70) lacking σ region 1.1 are not released during transcription initiation and elongation. Proc Natl Acad Sci USA. 2025;122:e2503801122. 10.1073/pnas.2503801122. 40961149 PMC12478191

[26] Doukhan L, Predich M, Nair G. et al. Genomic organization of the mycobacterial sigma gene cluster. Gene. 1995;165:67–70. 10.1016/0378-1119(95)00427-8. 7489918

[27] Gomez M, Doukhan L, Nair G. et al. sigA is an essential gene in *Mycobacterium smegmati*s. Mol Microbiol. 1998;29:617–28. 10.1046/j.1365-2958.1998.00960.x. 9720877

[28] Manganelli R, Provvedi R, Rodrigue S. et al. Sigma factors and global gene regulation in *Mycobacterium tuberculosis*. J Bacteriol. 2004;186:895–902. 10.1128/JB.186.4.895-902.2004. 14761983 PMC344228

[29] Koo B-M, Rhodius VA, Campbell EA. et al. Mutational analysis of *Escherichia coli* sigma28 and its target promoters reveals recognition of a composite −10 region, comprised of an ‘extended −10’ motif and a core −10 element. Mol Microbiol. 2009;72:830–43. 10.1111/j.1365-2958.2009.06691.x. 19400790 PMC2756079

[30] Koo B-M, Rhodius VA, Campbell EA. et al. Dissection of recognition determinants of *Escherichia coli* sigma32 suggests a composite -10 region with an ‘extended −10’ motif and a core −10 element. Mol Microbiol. 2009;72:815–29. 10.1111/j.1365-2958.2009.06690.x. 19400791 PMC4412615

[31] Koo B-M, Rhodius VA, Nonaka G. et al. Reduced capacity of alternative sigmas to melt promoters ensures stringent promoter recognition. Genes Dev. 2009;23:2426–36. 10.1101/gad.1843709. 19833768 PMC2764494

[32] Campagne S, Marsh ME, Capitani G. et al. Structural basis for −10 promoter element melting by environmentally induced sigma factors. Nat Struct Mol Biol. 2014;21:269–76. 10.1038/nsmb.2777. 24531660

[33] Lane WJ, Darst SA. The structural basis for promoter −35 element recognition by the group IV sigma factors. PLoS Biol. 2006;4:e269. 10.1371/journal.pbio.0040269. 16903784 PMC1540707

[34] Perumal AS, Vishwakarma RK, Morichaud Z. et al. RbpA relaxes promoter selectivity of *M. tuberculosis*. RNA Polymerase. 2018;46:10106–18. 10.1093/nar/gky714 PMC6212719 30102406

[35] Boyaci H, Chen J, Jansen R. et al. Structures of an RNA polymerase promoter melting intermediate elucidate DNA unwinding. Nature. 2019;565:382–5. 10.1038/s41586-018-0840-5. 30626968 PMC6399747

[36] Jensen D, Manzano AR, Rammohan J. et al. CarD and RbpA modify the kinetics of initial transcription and slow promoter escape of the *Mycobacterium tuberculosis* RNA polymerase. Nucleic Acids Res. 2019;47:6685–98. 10.1093/nar/gkz449. 31127308 PMC6648326

[37] Banerjee R, Rudra P, Prajapati RK. et al. Optimization of recombinant *Mycobacterium tuberculosis* RNA polymerase expression and purification. Tuberculosis. 2014;94:397–404. 10.1016/j.tube.2014.03.008. 24832563

[38] Chin JW, Santoro SW, Martin AB. et al. Addition of p-azido-L-phenylalanine to the genetic code of *Escherichia coli*. J Am Chem Soc. 2002;124:9026–7. 10.1021/ja027007w. 12148987

[39] Jacques J-F, Rodrigue S, Brzezinski R. et al. A recombinant *Mycobacterium tuberculosis in vitro* transcription system. FEMS Microbiol Lett. 2006;255:140–7. 10.1111/j.1574-6968.2005.00071.x. 16436073

[40] Chakraborty A, Mazumder A, Lin M. et al. Site-specific incorporation of probes into RNA polymerase by unnatural-amino-acid mutagenesis and Staudinger–Bertozzi ligation. Methods Mol Biol. 2015;1276:101–31. 10.1007/978-1-4939-2392-2_6. 25665560 PMC4677679

[41] Burian J, Ramón-García S, Sweet G. et al. The mycobacterial transcriptional regulator whiB7 gene links redox homeostasis and intrinsic antibiotic resistance. J Biol Chem. 2012;287:299–310. 10.1074/jbc.M111.302588. 22069311 PMC3249081

[42] Hu Y, Coates AR. Transcription of two sigma 70 homologue genes, sigA and sigB, in stationary-phase *Mycobacterium tuberculosis*. J Bacteriol. 1999;181:469–76. 10.1128/JB.181.2.469-476.1999. 9882660 PMC93400

[43] Kenney TJ, Churchward G. Genetic analysis of the *Mycobacterium smegmatis* rpsL promoter. J Bacteriol. 1996;178:3564–71. 10.1128/jb.178.12.3564-3571.1996. 8655555 PMC178127

[44] Master S, Zahrt TC, Song J. et al. Mapping of *Mycobacterium tuberculosis* katG promoters and their differential expression in infected macrophages. J Bacteriol. 2001;183:4033–9. 10.1128/JB.183.13.4033-4039.2001. 11395468 PMC95287

[45] Gonzalez-Y-Merchand JA, Colston MJ, Cox RA. The rRNA operons of *Mycobacterium smegmatis* and *Mycobacterium tuberculosis*: comparison of promoter elements and of neighbouring upstream genes. Microbiology. 1996;142–667–74. 10.1099/13500872-142-3-667. 8868442

[46] Song T, Song S-E, Raman S. et al. Critical role of a single position in the −35 element for promoter recognition by *Mycobacterium tuberculosis* SigE and SigH. J Bacteriol. 2008;190:2227–30. 10.1128/JB.01642-07. 18192397 PMC2258898

[47] Lee J-H, Karakousis PC, Bishai WR. Roles of SigB and SigF in the *Mycobacterium tuberculosis* sigma factor network. J Bacteriol. 2008;190:699–707. 10.1128/JB.01273-07. 17993538 PMC2223694

[48] Zhu DX, Stallings CL. Transcription regulation by CarD in mycobacteria is guided by basal promoter kinetics. J Biol Chem. 2023;299:104724. 10.1101/2023.03.16.533025. 37075846 PMC10232725

[49] Beuth B, Pennell S, Arnvig KB. et al. Structure of a *Mycobacterium tuberculosis* NusA–RNA complex. EMBO J. 2005;24:3576–87. 10.1038/sj.emboj.7600829. 16193062 PMC1276712

[50] Arnvig KB, Pennell S, Gopal B. et al. A high-affinity interaction between NusA and the rrn nut site in *Mycobacterium tuberculosis*. Proc Natl Acad Sci USA. 2004;101:8325–30. 10.1073/pnas.0401287101. 15159542 PMC420393

[51] Jayasinghe OT, Mandell ZF, Yakhnin AV et al. Transcriptome-wide effects of NusA on RNA polymerase pausing in *Bacillus subtilis*. J Bacteriol. 2022;204:e0053421. 10.1128/jb.00534-21. 35258320 PMC9112975

[52] Hubin EA, Fay A, Xu C. et al. Structure and function of the mycobacterial transcription initiation complex with the essential regulator RbpA. eLife. 2017;6:e22520 10.7554/eLife.22520. 28067618 PMC5302886

[53] Hu Y, Morichaud Z, Chen S. et al. *Mycobacterium tuberculosis* RbpA protein is a new type of transcriptional activator that stabilizes the σ A-containing RNA polymerase holoenzyme. Nucleic Acids Res. 2012;40:6547–57. 10.1093/nar/gks346. 22570422 PMC3413145

[54] Beaucher J, Rodrigue S, Jacques P-E. et al. Novel *Mycobacterium tuberculosis* anti-sigma factor antagonists control sigmaF activity by distinct mechanisms. Mol Microbiol. 2002;45:1527–40. 10.1046/j.1365-2958.2002.03135.x. 12354223

[55] Fang C, Li L, Shen L. et al. Structures and mechanism of transcription initiation by bacterial ECF factors. Nucleic Acids Res. 2019;47:7094–104. 10.1093/nar/gkz470. 31131408 PMC6648896

[56] Callaci S, Heyduk E, Heyduk T. Core RNA polymerase from *E. coli* induces a major change in the domain arrangement of the sigma 70 subunit. Mol Cell. 1999;3:229–38. 10.1016/S1097-2765(00)80313-5. 10078205

[57] Schwartz EC, Shekhtman A, Dutta K. et al. A full-length group 1 bacterial sigma factor adopts a compact structure incompatible with DNA binding. Chem Biol. 2008;15:1091–103. 10.1016/j.chembiol.2008.09.008. 18940669 PMC2677525

[58] Joron K, Zamel J, Kalisman N. et al. Evidence for a compact σ(70) conformation *in vitro* and *in vivo*. iScience. 2024;27:110140. 10.1016/j.isci.2024.110140. 38957792 PMC11217687

[59] Dombroski AJ, Walter WA, Gross CA. Amino-terminal amino acids modulate sigma-factor DNA-binding activity. Genes Dev. 1993;7:2446–55. 10.1101/gad.7.12a.2446. 8253389

[60] Dombroski AJ, Walter WA, Record MTJ. et al. Polypeptides containing highly conserved regions of transcription initiation factor sigma 70 exhibit specificity of binding to promoter DNA. Cell. 1992;70:501–12. 10.1016/0092-8674(92)90174-B. 1643661

