# Supplementary material for: Differential association of primary and alternative σ factors with RNA polymerase of Mycobacterium tuberculosis during transcription elongation
Source: Nucleic Acids Res. 2026 Jan 8;54(1):gkaf1459. doi: 10.1093/nar/gkaf1459 (PMC12781872; doi:10.1093/nar/gkaf1459)
Supplement: gkaf1459_Supplemental_File [file gkaf1459_supplemental_file.pdf]

Supplementary Information for

Differential Association of Primary and Alternative σ Factors with RNA Polymerase of *Mycobacterium Tuberculosis* during Transcription Elongation

AUTHORS

Nilanjana Hazra[1] and Jayanta Mukhopadhyay[1*]

[1]Department of Chemical Sciences, Bose Institute, EN 80, Sector V, Bidhan Nagar, Kolkata – 700091, India

*To whom correspondence should be addressed. Tel: +919007015557; Email: jayanta@jcbose.ac.in

Corresponding author: Jayanta Mukhopadhyay

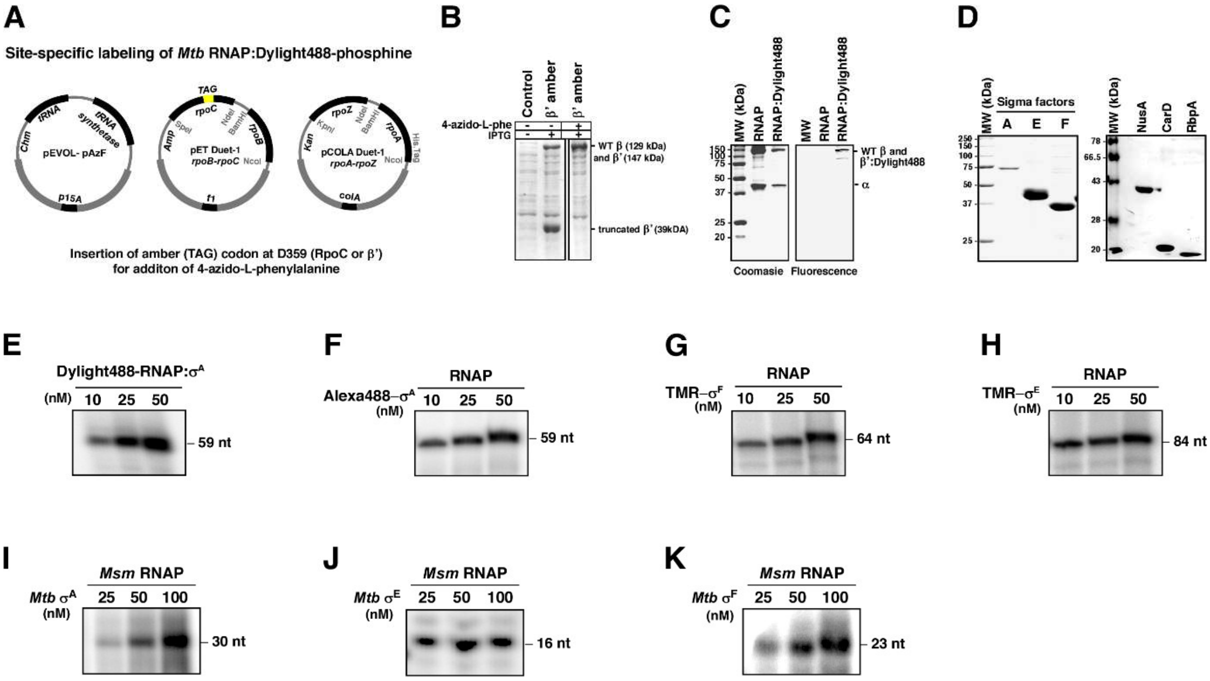

Supplementary Figure S1. A, Combination of plasmid constructs used to co-transform *E. coli* BL21 (DE3) cells for azide-specific labelling of β' subunit of RNAP. **B**, The incorporation of 4-azido-L-phenylalanine at the substituted TAG codon was validated using 10% SDS PAGE. **C**, Dylight-488 labelled RNAP core was resolved on 10% SDS PAGE and visualised using Amersham Typhoon imager using Dylight-488 channel. **D**, Purity of *Mtb* σ factors (σ^A, σ^E and σ^F) and transcription factors (NusA, CarD and RbpA). **E-H**, *In vitro* transcription assay with labelled proteins (Dylight488-RNAP, Alexafluor488-σ^A, TMR-σ^F and TMR-σ^E). **I-K**, *In vitro* transcription assay to confirm the ability of *Mtb* σs to function with *Msm* RNAP.

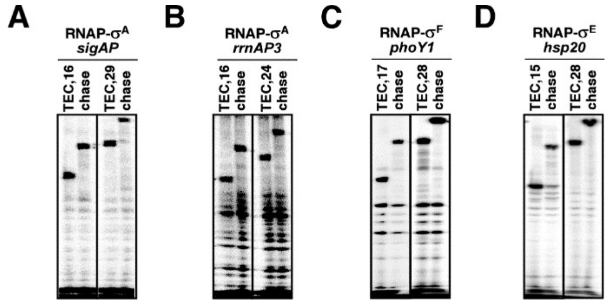

Supplementary Figure S2. *In vitro* transcription assay to visualize RNA from stalled TECs on alternate promoters. A-D, RNAP and respective σs were incubated with template DNA (*sigAP*, *rrnAP3*, *phoY1* and *hsp20*) to form RPo. Stalled TEC_{+n} was formed by the addition of 3 NTPs as mentioned in materials and methods section. The samples were resolved using 15% Urea PAGE.

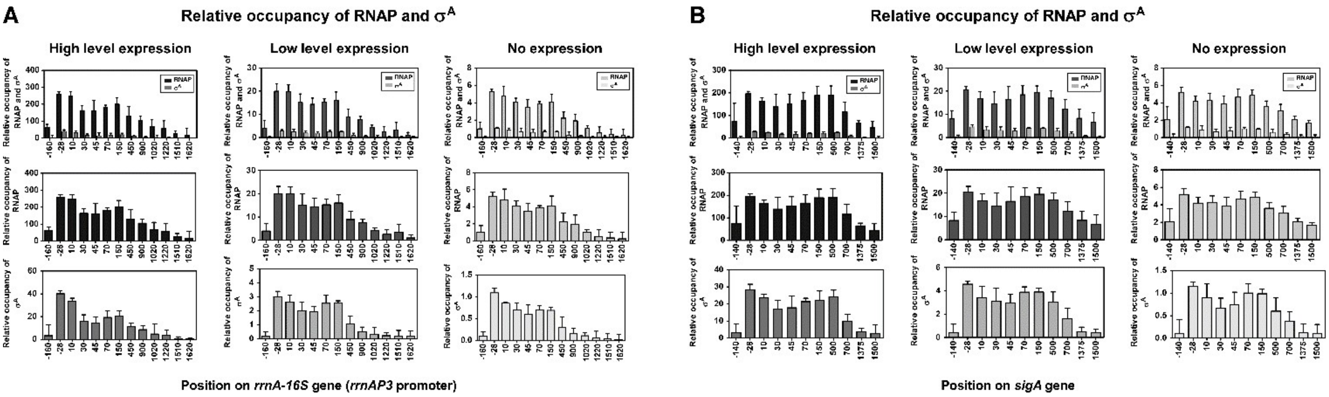

Supplementary Figure S3. Relative occupancies of RNAP and σ^A without normalization. **A**, Relative occupancies ($E^{-\Delta Ct}$) of RNAP (pulled down by anti-β antibody) and σ^A on *Msm* 16S *rrnA* gene, obtained from ChIP-qPCR analysis under high, low and no expression of *Mtb*.σ^A. **B,** As A, except *on sigA* gene. Each bar represents a mean of 3 independent replicates with standard deviations represented as error bars.

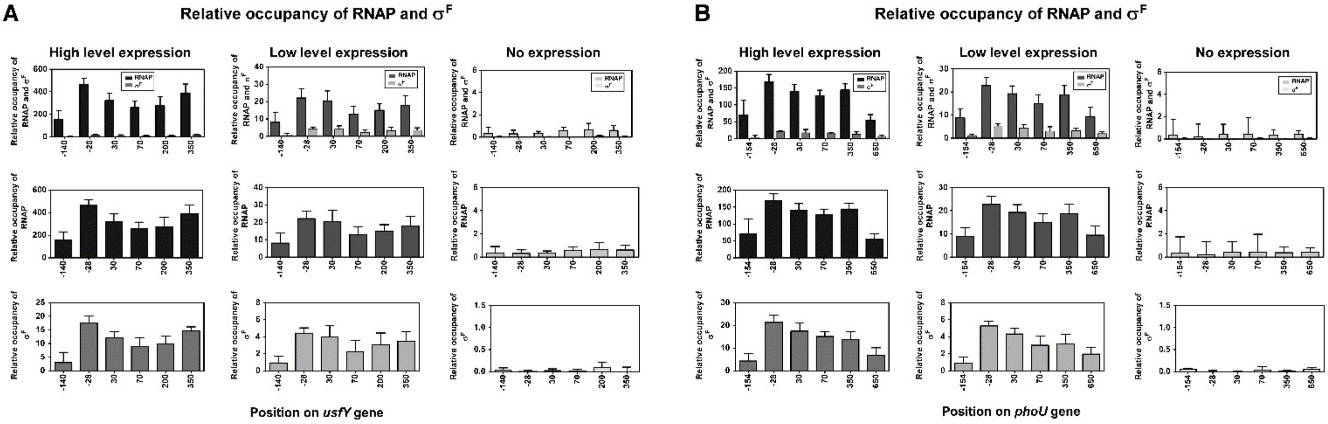

Supplementary Figure S4. Relative occupancies of RNAP and σ^F without normalization. **A-B,** Relative occupancies ($E^{-\Delta Ct}$) of RNAP and σ^F on *Msm usfY* and *phoU* genes under high, low and no expression of *Mtb* σ^F. Each bar represents a mean of 3 independent replicates with standard deviations represented as error bars.

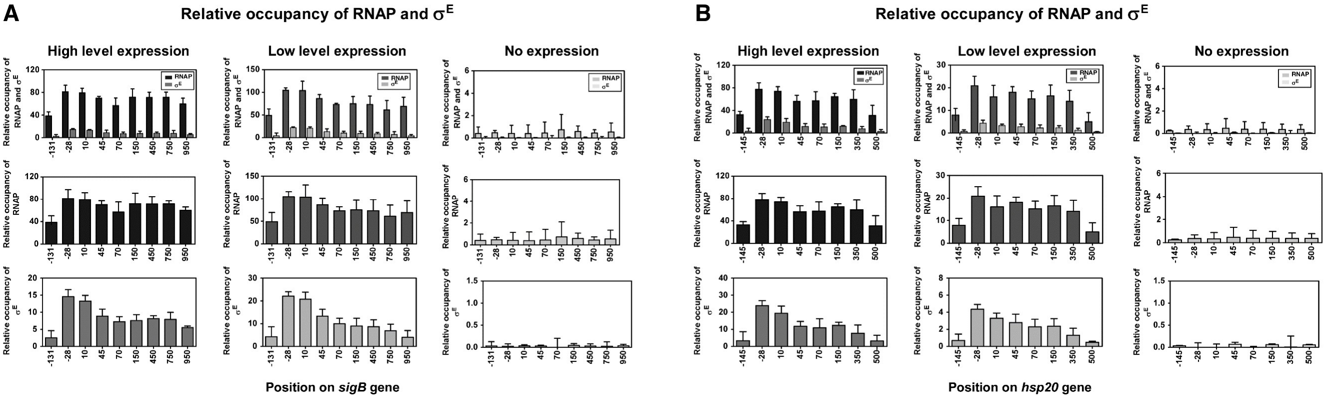

Supplementary Figure S5. Relative occupancies of RNAP and σ^E without normalization. **A-B,** Relative occupancies ($E^{-\Delta Ct}$) of RNAP and σ^E on *Msm sigB* and *hsp20* genes obtained from ChIP-qPCR analysis under high, low and no expression of *Mtb* σ^E.Each bar represents a mean of 3 independent replicates with standard deviations represented as error bars.

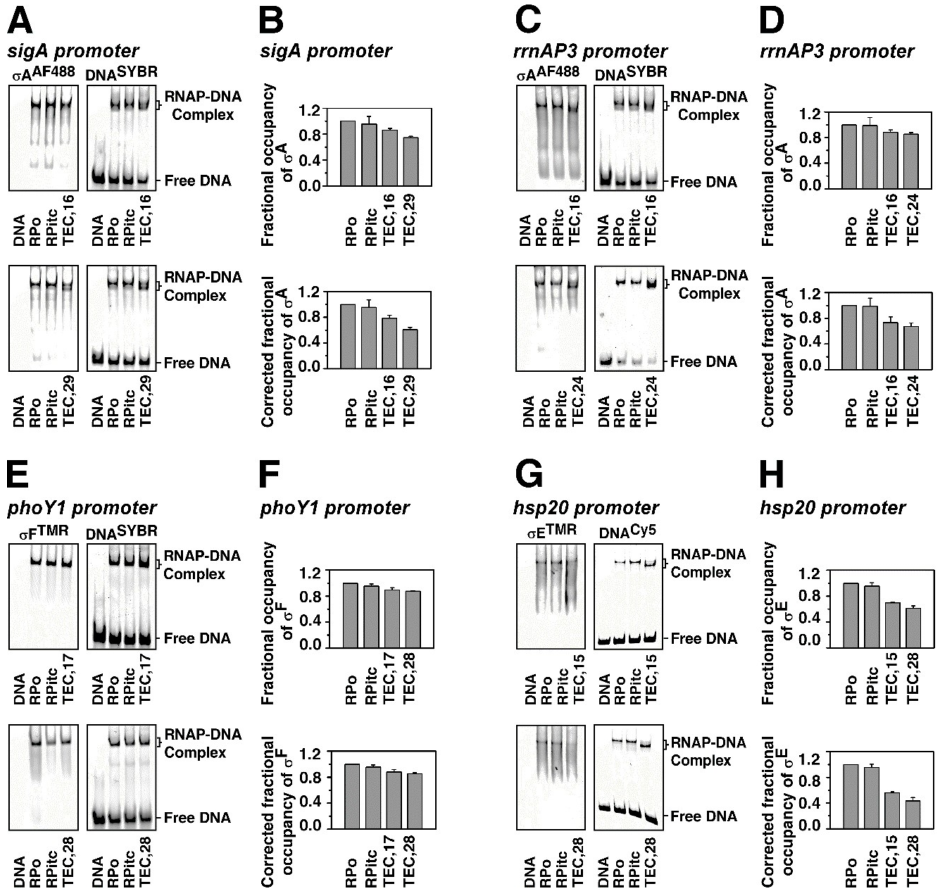

Supplementary Figure S6. Occupancy of σ retained on TECs during elongation on alternate promoters: EMSA assays. A, RNAP and Alexafluor-488 labelled σ^A was incubated with unlabelled *sigA* template having pause sites at n+1 (n=16 and 29). TEC stalls at respective positions after addition of 3 NTPs. The gel was scanned for fluorescence (488 channel: left panel shows Alexafluor-488 labelled σ^A; TMR channel: right panel shows DNA stained with SYBR gold post 488 scan). **B**, Upper panel: Fractional occupancies of σ^A with respect to TEC on *sigA*, determined as the ratio of fluorescence signal of σ^A and that of DNA at RPo, RPitc and TEC_{+n} from EMSA. Lower panel: Fractional occupancy of σ^A on *sigA* at RPitc and TEC_{+n}, after correcting for the subpopulation of RNAP complexes at RPo, incompetent to form TECs. **C-D,** Same as A-B, except *rrnAP3* template was used to stall TEC at n+1 (n=16 and 24) sites, post TSS. **E-F,** As A-B, except RNAP and TMR labelled σ^F was incubated with unlabelled *phoY1* template with pause sites at n+1 (n=17 and 28). TECs formed after 3 NTP addition, stalling at respective TEC_{+n} is scanned for fluorescence (TMR channel: left panel shows TMR labelled σ^F; 488 channel: right panel shows DNA stained with SYBR gold post TMR scan). **G-H,** As A-B, except RNAP and TMR labelled σ^E were incubated with 5' Cy5 labelled *hsp20* template having pause site at n+1 (n=15 and 28). RNAP stalls at the n+1 position after addition of 3 NTPs. Gels are scanned for fluorescence (TMR channel for σ^E and Cy5 channel for DNA). Each bar represents a mean of 3 independent replicates with standard deviations represented as error bars.

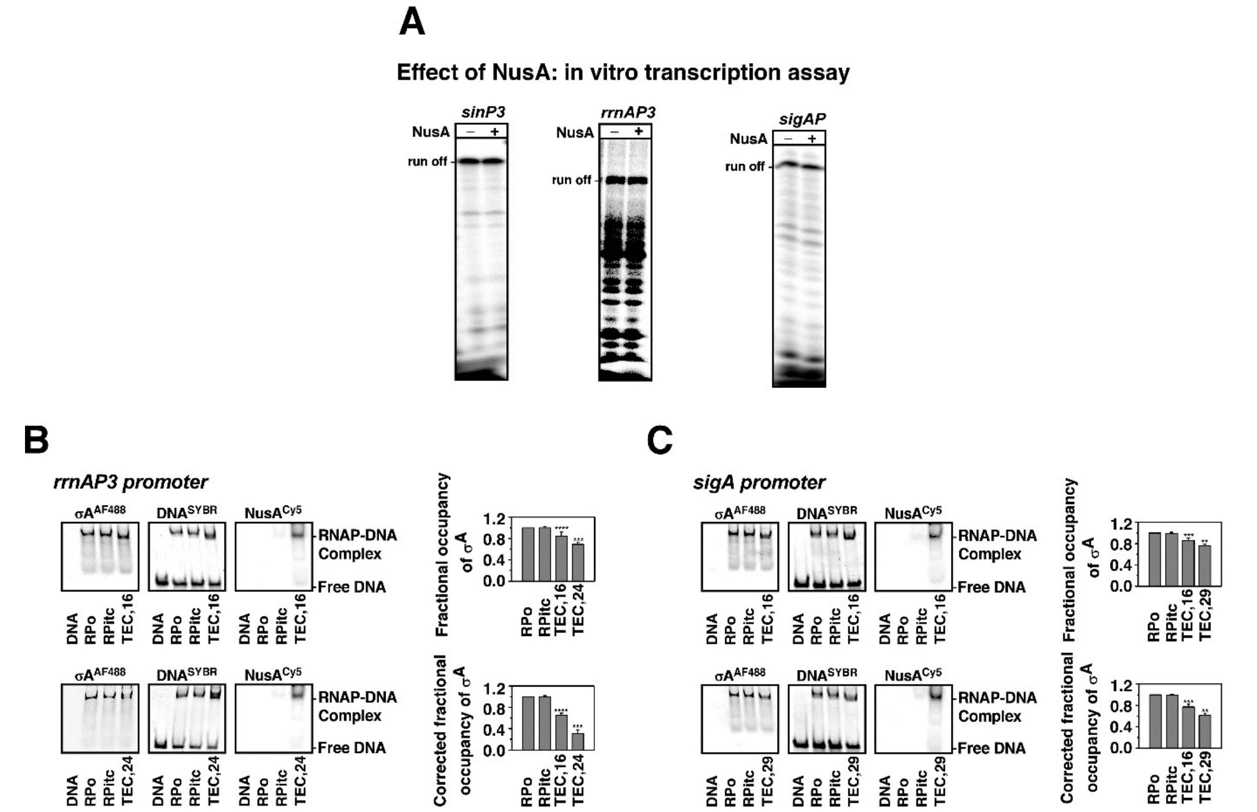

Supplementary Figure S7. Effect of NusA on σ^A-release from TECs: IVT and EMSA assay. A, No effect of NusA on transcription of RNAP-σ^A on *sinP3*, *rrnAP3* and *sigAP* DNA templates. **B,** As C-D from *Supplementary Figure S6* but in the presence of NusA on *rrnAP3* template. **C**, As A-B from *Supplementary Figure S6* but in the presence of NusA on *sigA* template. Each bar represents a mean of 3 independent replicates with standard deviations represented as error bars. (**, ***, **** denotes p<0.01, 0.005 and 0.001, respectively).

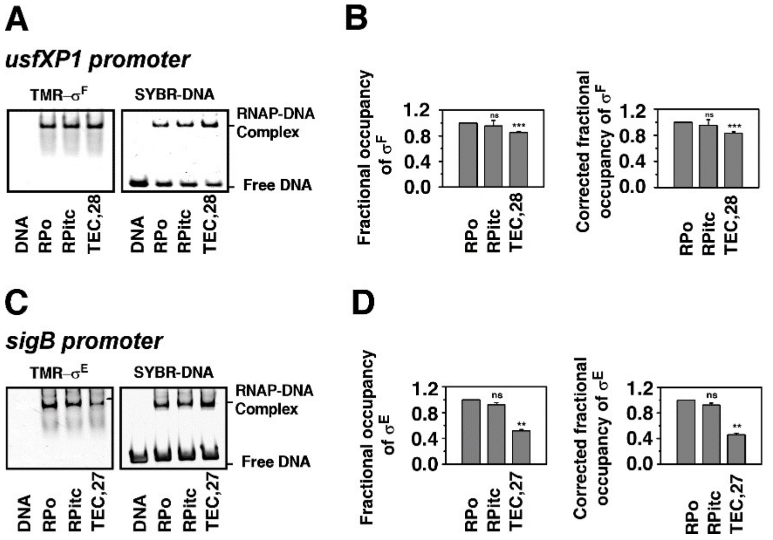

Supplementary Figure S8. Effect of NusA on σ-release (σ^F and σ^E) from TECs: EMSA assay. A-B, As C-D from Figure 3, on TEC$_{+28}$, in the presence of NusA. **C-D,** As H-I from Figure 3, on TEC$_{+27}$, in the presence of NusA. Each bar represents a mean of 3 independent replicates with standard deviations represented as error bars. (**, *** denotes p<0.01, and 0.005 respectively; ns denotes non-significance).

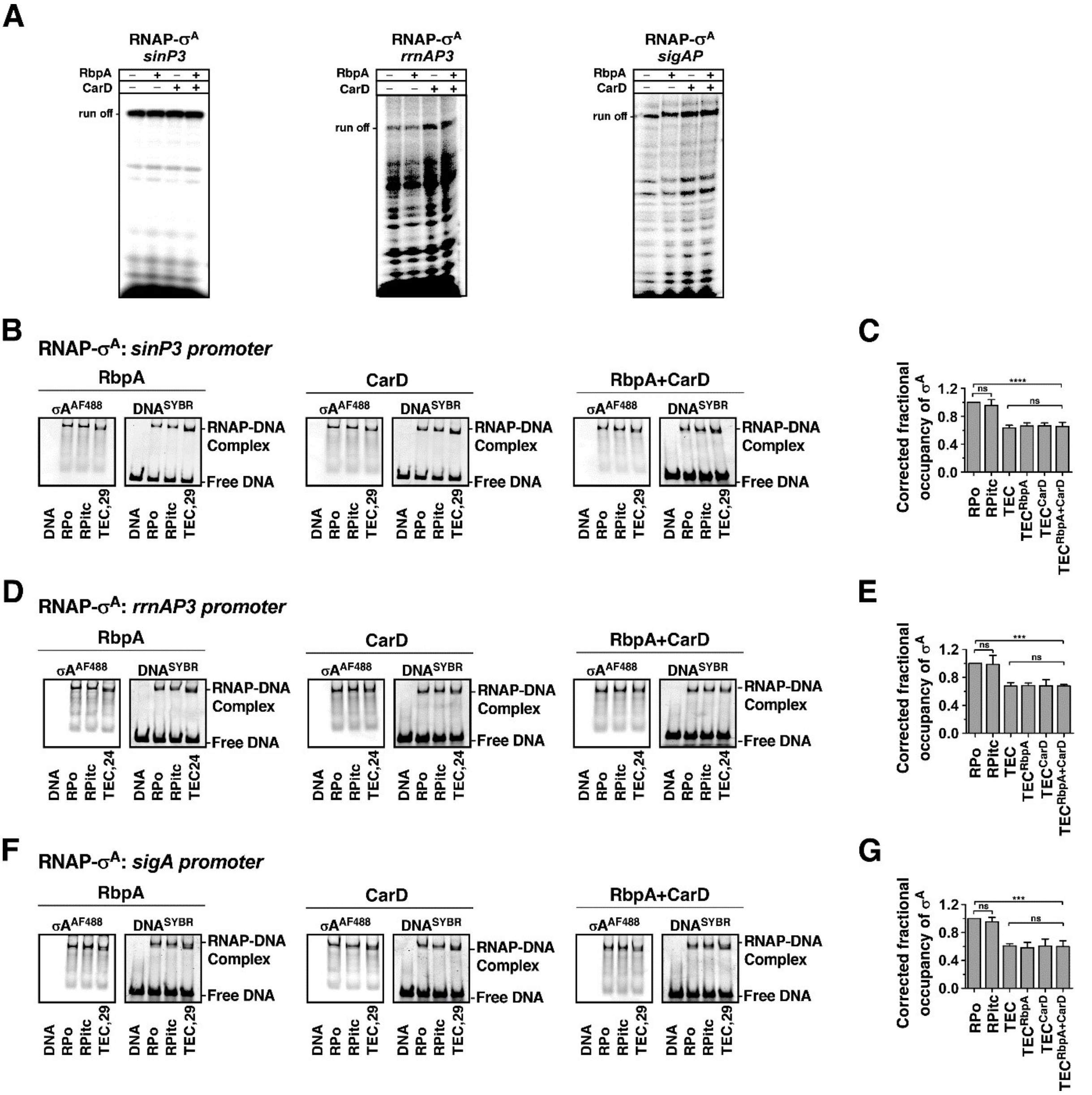

Supplementary Figure S9. Effect of RbpA/CarD on σ^A-release from TECs: IVT and EMSA assay. **A,** No effect of RbpA or CarD on transcription of RNAP-σ^A on *sinP3* DNA template. Presence of CarD improved transcription yield on *rrnAP3* and *sigAP* template DNA. **B-C,** As Figure 2C-D, except RbpA, CarD or RbpA and CarD were added to monitor effect on σ^A release. **D-G**, As *Supplementary Figure S6A-D*, except RbpA, CarD or RbpA and CarD were added to monitor effect on σ^A release. Each bar represents a mean of 3 independent replicates with standard deviations represented as error bars; statistical significance was assigned based on p-value <0.05 (***, **** denotes p<0.005 and 0.001, respectively; ns denotes non-significance).

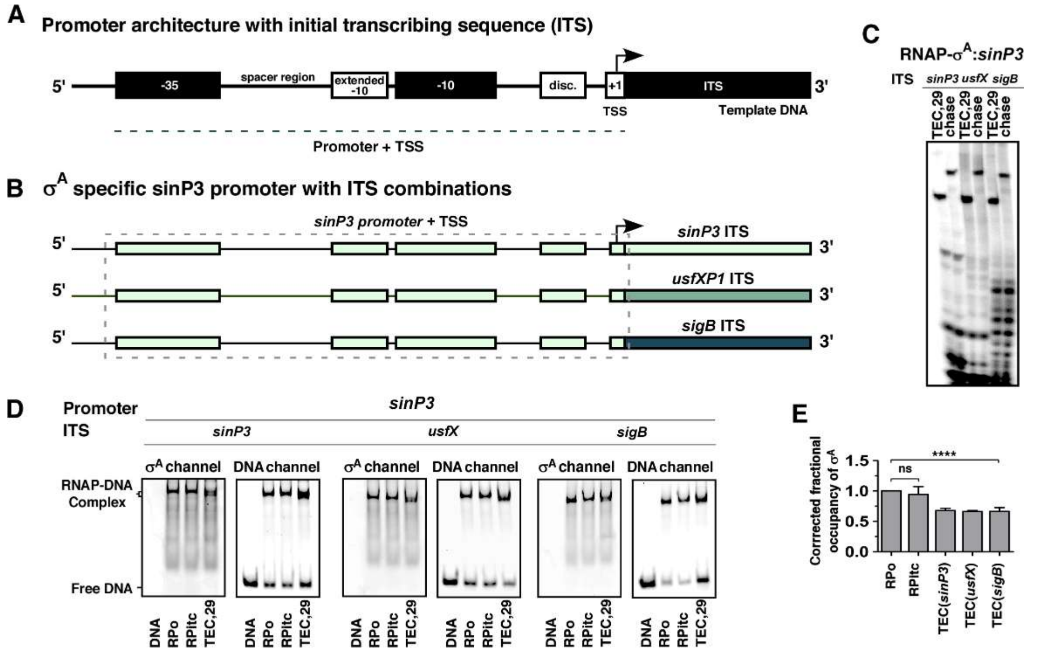

Supplementary Figure S10. Effect of initial transcribing sequences (ITS) on σ^{A}-release from TECs: IVT and EMSA assay. A, Schematic representation of general promoter and template DNA architecture. The promoter comprises -35 element, -10 element, extended -10 element, a spacer region and the +1 TSS. ITS comprises the initial transcribing sequences of the specific gene located immediately downstream to the TSS. **B**, Schematic representation of DNA constructs with *sinP3* promoter and various ITS sequences (*sinP3*, *usfX* and *sigB*). **C**, *In vitro* transcription shows RNAP-σ^{A} mediated transcription on these DNA constructs with various ITS. **D-E,** EMSA assays showing σ^{A} occupancies on TECs with various ITS sequences. Each bar represents a mean of 3 independent replicates with standard deviations represented as error bars; statistical significance was assigned based on p-value <0.05 (**** denotes p<0.001; ns denotes non-significance).

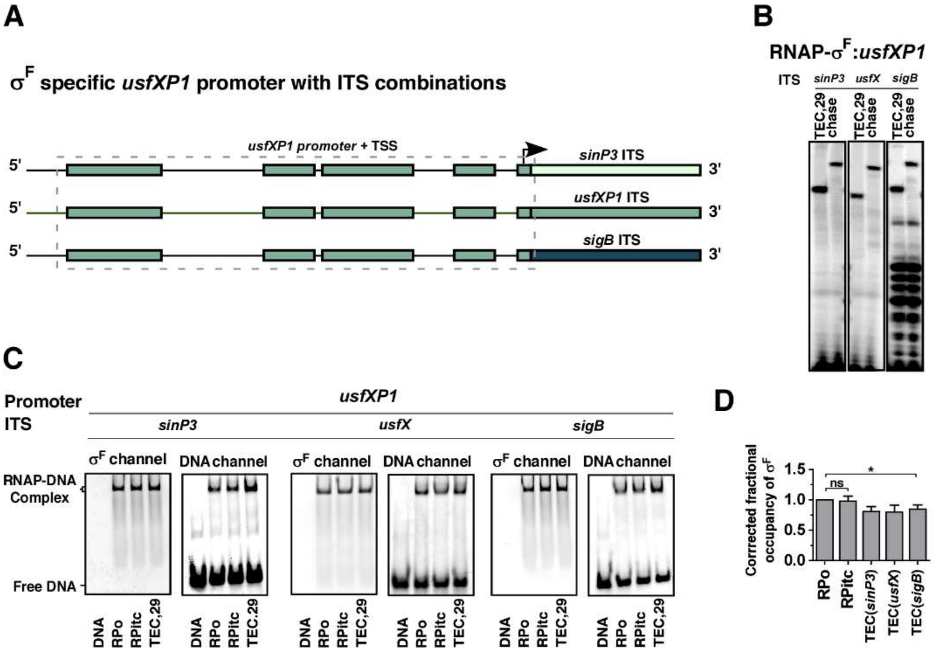

Supplementary Figure S11. Effect of initial transcribing sequences (ITS) on σ^F-release from TECs: IVT and EMSA assay. A, Schematic representation of DNA constructs with *usfXP1* promoter and various ITS sequences (*sinP3*, *usfX* and *sigB*). **B**, *In vitro* transcription shows RNAP-σ^F mediated transcription on these DNA constructs with various ITS. **C-D,** EMSA assays showing σ^F occupancies on TECs with various ITS sequences. Each bar represents a mean of 3 independent replicates with standard deviations represented as error bars; statistical significance was assigned based on p-value <0.05 (*denotes p<0.05; ns denotes non-significance).

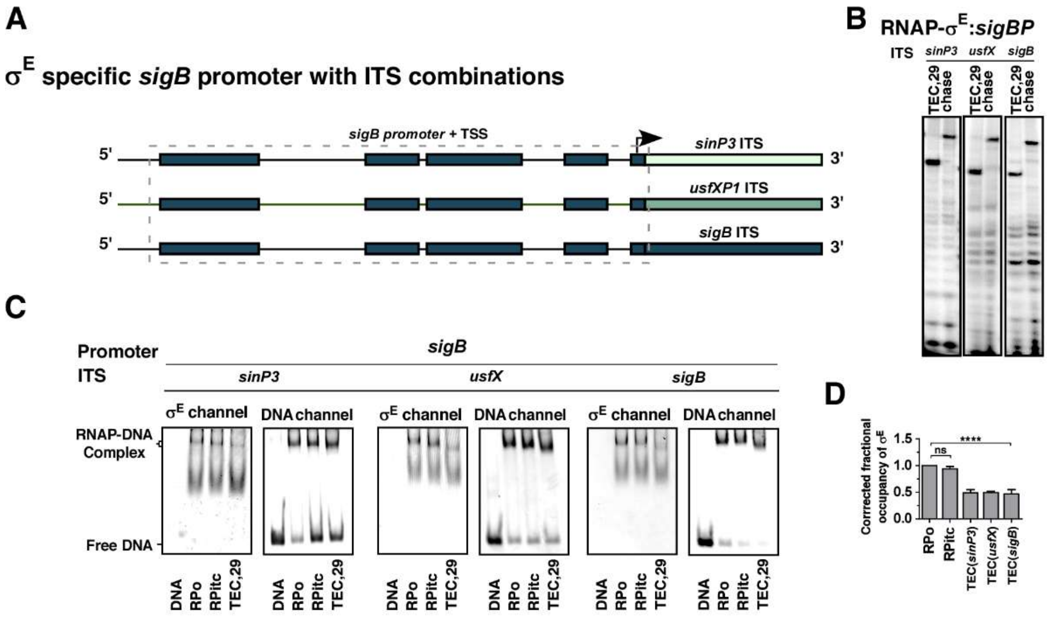

Supplementary Figure S12. Effect of initial transcribing sequences (ITS) on σ^{E}-release from TECs: IVT and EMSA assay. A, Schematic representation of DNA constructs with *sigB* promoter and various ITS sequences (*sinP3*, *usfX* and *sigB*). **B**, *In vitro* transcription shows RNAP-σ^{E} mediated transcription on these DNA constructs with various ITS. **C-D,** EMSA assays showing σ^{E} occupancies on TECs with various ITS sequences. Each bar represents a mean of 3 independent replicates with standard deviations represented as error bars; statistical significance was assigned based on p-value <0.05 (**** denotes p<0.001; ns denotes non-significance).

Supplementary Table S1. List of plasmid constructs used in this study

Name of plasmid	Important features	Used In
pET Duet-1 *rpoB-rpoC*	*Mtb* RpoB and RpoC	Banerjee *et al.*(1)
pCOLA Duet-1 *rpoA-rpoZ*	*Mtb* RpoA and RpoZ	This study
pEVOL-pAzF	tRNA, tRNA synthetase for pAzF incorporation	Schultz *et al.* (2)
Mutated pET Duet-1 *rpoB-rpoC* mut	D359Amber in *Mtb* RpoC protein, *rpoC*-TAGxTAA	This study
Mutated pCOLA Duet-1 *rpoA-rpoZ*	*Mtb rpoA*-TAGxTAA, *rpoZ*-TAGxTAA	This study
pET Duet-1 σ^{A}	*Mtb* σ^{A}	This study
pET30a σ^{E}	*Mtb* σ^{E}	Rodrigue *et al.*(3)
pET30a σ^{F}	*Mtb* σ^{F}	Rodrigue *et al.*(3)
pET30a σ^{F}-σ_3 mut	*Mtb* σ^{F} σ_3 A156C	This study
pET30a σ^{F}-σ_4 mut	*Mtb* σ^{F} σ_4 A235C	This study
pETDuet-1 σ^{A}-σ_2 mut	*Mtb* σ^{A} σ_2 A331C	This study
pETDuet-1 σ^{A}-σ_3 mut	*Mtb* σ^{A} σ_3 A400C	This study
pETDuet-1 σ^{A}-σ_4 mut	*Mtb* σ^{A} σ_4 A472C	This study
pET30a σ^{E} σ_2 mut	*Mtb* σ^{E} σ_3 A92C	This study
pACYC Duet-1 *nusA*	*Mtb* NusA	This study
pET28a *rbpA*	*Mtb* RbpA	This study
pET Duet-1 *carD*	*Mtb* CarD	This study
pLAM12 σ^{A}	*Mtb* σ^{A}	This study
pLAM12 σ^{E}	*Mtb* σ^{E}	This study
pLAM12 σ^{F}	*Mtb* σ^{F}	This study

Supplementary Table S2. Oligonucleotides used for cloning of *Mtb* genes

Name of the primer	Sequence (5' → 3')
NusA EcoRI FW	AGTACCGGAATTCGATGAACATCGACATGGCTGCTCTGC
NusA HindIII RV	GACGTCCCAAGCTTCTAGCGGTCGTGCGCCATAC
CarD BamHI FW	CACAGCCAGGATCCGATGATTTTCAAGGTCGGAGACACC
CarD KpnI RV	ACTCGAGGGTACCTCAAGACGCGGCGGCTAAAAC
σ^{A} NdeI PLAM12 FW	CCCATATGGTGGCAGCGACCAAAGCAAGCACGG
σ^{A} HindIII PLAM12 RV	CCCAAGCTTGGGTCAGTCCAGGTAGTCGCGCAG
σ^{E} NdeI PLAM12 FW	CCCATATGATGGAACTCCTCGGCGGACCCC
σ^{E} EcoRI PLAM12 RV	AAAGCTTGAATTCTCAGCGAACTGGGTTGACGTGAACTGC

Supplementary Table S3. Oligonucleotides used for mutagenesis on *Mtb* genes

Name of the primer	Sequence (5’ → 3’)
RpoC D359AMBER FW	GAAAAGGCTGATCTAGCTGGGTGCGCCG
RpoC D359AMBER RV	CGGCGCACCCAGCTAGATCAGCCTTTTC
RpoC TAGXTAA FW	CAGCGACTACCGCTAAGATATCGGGGGATCC
RpoC TAGXTAA RV	GGATCCCCCGATATCTTAGCGGTAGTCGCTG
RpoA TAGXTAA FW	CGCCGAAACCGAACAGCTTTAAAATTCGAATTCGAGCTCGG
RpoA TAGXTAA RV	CCGAGCTCGAATTCGAATTTTAAAGCTGTTCGGTTTCGGCG
RpoZ TAGXTAA FW	CCGAGGGCGAGTAAGGTACCCTCGAGTC
RpoZ TAGXTAA RV	GACTCGAGGGTACCTTACTCGCCCTCGG
σ^F σ_3 A156C FW	GCGGAGCTCGGGATGGACCGCTGTGAGGTTATCGAAGGTTTGCTG
σ^F σ_3 A156C RV	CAGCAAACCTTCGATAACCTCACAGCGGTCCATCCCGAGCTCCGC
σ^F σ_4 A235C FW	GACTCGATGACCCAAACGCAGATCTGCGAGCGCGTCGGTATCTCACAG
σ^F σ_4 A235C RV	CTGTGAGATACCGACGCGCTCGCAGATCTGCGTTTGGGTCATCGAGTC
σ^A σ_2 A331C FW	CTGGGGCTGATCCGCTGCGTGGAGAAGTTCGAC
σ^A σ_2 A331C RV	GTCGAACTTCTCCACGCAGCGGATCAGCCCCAG
σ^A σ_3 A400C FW	CCACGCCCGAGGAGCTGTGCAAAGAGATGGACATCACCC
σ^A σ_3 A400C RV	GGGTGATGTCCATCTCTTTGCACAGCTCCTCGGGCGTGG
σ^A σ_4 A472C FW	CTCTCCGAGCGTGAGTGCGGCGTGGTGCGGCTAC
σ^A σ_4 A472C RV	GTAGCCGCACCACGCCGCACTCACGCTCGGAGAG
σ^E σ_2 A92C FW	GGTCCGTCAGCACTGCGATCGGGTGTACCG
σ^E σ_2 A92C RV	CGGTACACCCGATCGCAGTGCTGACGGACC

Supplementary Table S4. Sequences of DNA fragments used for *in vitro* transcription assay and EMSA assays

Name of promoter	DNA Sequence (5’ → 3’)
Bsu sinP3	GTGGAATTGTGACGGATAACAATTTCACGAATTCCAGCCAGAAGTCATAC<br>CGTAAATCCTTTCTGAATGTGC**TATAAT**ATCACA**A**TTGCTCGATGAGAAA<br>CATGAAACCGAATACGGATCCTCTAGAGTCGACCTGCAGGCATGCAAGC<br>TTGGC
	**+17C:** ATTGGTGGATGAGAAACATGAAAGGGAAT
	**+24C:** ATTGGTGGATGAGAAAGATGAAACGGAATAGGGA
	**+30C:** ATTGGTGGATGAGAAAGATGAAAGGGAATCGGGATGGTGTA
	**+60C:**<br>ATTGGTGGATGAGAAAGATGAAAGGGAATAGGGATGGTGGAGAGG<br>GGAGGTGGAGGGATCGAAGGTTGGG
Mtb rrnAP3	GTTAGGCGACGGTCACCTATGGATATCTATGGATGACCGAACCTGGTC**TT**<br>**GACT**CCATTGCCGGATTTGTAT**TAGACT**GGCAGG**G**TCGCCCCGAAGCGG<br>GCGGAAACAAGCAAGCGTGTTGTTTGAGAACTCAATAGTGTGTTTGGTG<br>GTTTCACATTTTTGTTGTTATTTTTGGCCATGCTCTTGATGCCCCGTTGTC<br>GGGGGCGTGGCCGTTTGTTTTGTCAGGATATTTCTAAATACCTTTGGCTC<br>CCTTTTCCAAAGGGAGTGTTTGGGTTTTGTTTGGAGAGTTTGATCCTGGC<br>TCAGGACGAACGCTGGCGGCGTAGGATCCAGAGG
	**+11A:** GTCGCCCCGTAGCGGGCGGAA
	**+17A:** GTCGCCCCGTTGCGGGAGGAAACAAGC
	**+25A:** GTCGCCCCGTTGCGGGCGGTTTCTAGCCAGCGTGT
Mtb sigA	GGCCATGGCCTCTGTCGCAGATACGACGCACTGAAACTTGCCCGCTCGG<br>GC**TGTACT**CGTGCGCAGTAAAGT**TACAAT**GGTCAG**C**GGCGGCCGCCCG<br>ACCGATAGCGCGCGAGTATTCACGCTGATATCAACGCCGACATTCGACA<br>TAGCAGACACTTTCGGTTACGCACGCCCAGACCCAACCGGAAGTGAGTA<br>ACGACCGAAGGGGTGTATGTGGCAGCGACCAAAGCAAGCACGGCGACC<br>GATGAGCCGGTAAAACGCACCGCCACCAAGTCGCCCGCGGCTTCCGCG<br>TCCGGGGCCAAGACCGGCGCCAAGCGAACAGCGGCGAAGTCCGCTAGT<br>GGCTCCCCACCCGCGAAGCGGGCTACCAAGCCCGCGGCCCGGTCCGTC<br>AAGCCCGCCTCG

	**+17T:** CGGCGGCCGCCCGACCTATAGCGCGCG
	**+30T:** CGGCGGCCGCCCGACCGAAAGCGCGCGAGTATTCACGCTG
Mtb sigB	GTCTTCGGCAGATTCCTGCACGTCACAGGGCGTCAGATCACTGCTGGGT<br>**GGGAAC**TCAAAGTCCGGCTTTGT**CGTT**AAACCCCAT**G**ACAGTGCAAGCC<br>GATCGGGAGGTCGCTATGGCCGATGCACCCACAAGGGCCACCACAAGC<br>CGGGTTGACAGCGATCTGGATGCTCAAAGCCCCGCGGCGGACCTCGTG<br>CGCGTCTATCTGAACGGCATCGGCAAGACGGCGTTGCTCAACGCCGCC<br>GGTGAAGTCGAACTGGCCAAGCGCATAGAAGCCGGGTTGTATGCCGAG<br>CATCTGCTGGAAACCCGGAAGCGCCTCGGCGAGA
	**+16T:** GACAGCGCAAGCCGATCGGGAGGTCG
	**+24T**: GACAGCGCAAGCCGACCGGGAGGTCGCTATGGCC
	**+28T:** GACAGCGCAAGCCGACCGGGAGGCCGCTATGGCCGATG
	**+85T:** GACAGCGCAAGCCGACCGGGAGGCCGCCACGGCCGACGCACCC<br>ACAAGGGCCACCACAAGCCGGGCCGACAGCGACCCGGACGCTCAAAGC<br>CCCG
Mtb hsp20	ACCTGGCGACCGGCTGTGTGACAGTGTTCGCTTCCGGTGAACGCCGAG<br>GT**GGAACT**TAAGCGTGGTCGACTCAG**GTT**CTTGGTAT**A**GCGGGCCGGCA<br>ACGCGCCGGACACAGCTAACTCACAACGAAGCATGAGGAGATAACCATG<br>AACAATCTCGCATTGTGGTCGCGTCCGGTGTGGGACGTTGAGCCCTGGG<br>ACCGCTGGCTACGTGACTTCTTCGGCCCTGCCGCGACGACGGACTGGTA<br>CCGCCCGGTCGCCGGAGACTTCACGCCGGCCGCCGAGATCGTCAAGGA<br>TGGCGAC
	**+16T:** AGCGGGCCGGCGACGTGCCGGACACA
	**+29T:** AGCGGGCCGGCGACGCGCCGGACACAGCTAAGTCACAAC
Mtb usfXP1	GCTGGCCGGCTAGACATCCTAGTGCGGCTGGAAATCCCGGCATCGCG**G**<br>**GGTTT**CACCGGCAGCTGCGAAT**GGGTAT**CACGGGTA**C**ACCATGATGAAT<br>CCCGACCATGTTGCGTTAGATCCCCACTACCAGCAGGTCCGACCATGAC<br>CGACCAGCTCGAAGACCAGACCCAAGGCGGGAGTACTGTCGATCGAAG<br>CTTGCCGGGAGGGTGCATGGCCGACTCGGATTTACCCACCAAGGGGCG<br>CCAACGCGGTGTCCGCGCCGTCGAGCTG
	**+18T:** CACCACGACGAACCGCGTCCATGTTGCG
	**+24T:** CACCACGACGAACCGCGACCACGTTGCGTTAGAT
	**+29T:** CACCACGACGAACCGCGACCACGCCGCGTTAGATCCCCA
	**+65T:** **C**ACCACGACGAACCGCGACCACGCCGCGCCAGACCCCCACC<br>ACCAGCAGGCCCGACCACGACCGTCCAGCTCGAA
Mtb phoY1	CGGGCCAGCGCACCCGAGGCGCGTGCCGAGATCCTCGAGCCGGTGCC<br>TTT**GGATTG**AAGGCTACGCCAAGC**GGGTAT**GTCGCGCG**G**TGCCGCCAT<br>GGTCGATGATGGCGGTGTGGATCCGCGGCAGCACCACACGACGACTCA<br>GGGTCAGAGAGGCGTGACACCATGCGGACGGTCTATCA
	**+18A:** GTGCCGCCCTGGTCGCTATTGGCGGTGT
	**+29A:** GTGCCGCCCTGGTCGCTGTTGGCGGTGTAGATCCGCGGC

*Transcription start sites are highlighted in green, and RNAP pause sites are highlighted in red. Promoter -10 region is in bold and -35 region is underlined.

Supplementary Table S5. Oligonucleotides for the amplification of long templates used for *in vitro* transcription assay

Name of the primer	Sequence (5' → 3')
Bsu sinP3 FW	GTGGAATTGTGACGGATAACAATTTCACGAATTCCAGCCA
Bsu sinP3 +60C-i RV	CTCCACCTCCACTCTACACCATCCCTATTCCCTTTCATC
Bsu sinP3 +60C-ii RV	CCCAACCTTCGATCCCTCCACCTCC
Mtb sigBP FW	GTCTTCGGCAGATTCCTGCACGTCACAGGGCGTCAGATC
Mtb sigBP +85T-i RV	GGGTGCGTCGGCCGTGGCGGCCT
Mtb sigBP +85T-ii RV	GGCTTGTGGTGGCCCTTGTGGGTGC
Mtb sigBP +85T-iii RV	GGGTCGCTGTCGGCCCGGCTTGTG
Mtb sigBP +85T-iv RV	CGGGGCTTTGAGCGTCCGGGTCG
Mtb usfXP1 FW	GCTGGCCGGCTAGACATCCTAGTGCGGCTGGA
Mtb usfXP1 +65T-i RV	GGTGGGGGTCTGGCGCGGCGTG
Mtb usfXP1 +65T-ii RV	GTGGTCGGGCCTGCTGGTGGTGGG
Mtb usfXP1 +65T-iii RV	TTCGAGCTGGACGGTCGTGGTCGGG

Supplementary Table S6. The list of 5' Cy5-tagged oligonucleotides used to amplify DNA for the EMSA assays

Name of the primer	Sequence (5' → 3')
5' Cy5 *sinP3* FW	GTGGAATTGTGACGGATAACAATTTCACGAATTCCAGCCA
5' Cy5 *sigBP* FW	GTCTTCGGCAGATTCCTGCACGTCACAGGGCGTCAGATC
5' Cy5 *usfXP1* FW	GCTGGCCGGCTAGACATCCTAGTGCGGCTGGA
5' Cy5 *hsp20* FW	ACCTGGCGACCGGCTGTGTGA

Supplementary Table S7. Transcription efficiencies (f) calculated for the promoters of each σ factor

σ Factor	Promoter	Transcription Efficiency (f)
σ^A	***sinP3***	0.85
	sigA	0.63
	rrnAP3	0.45
σ^F	***usfXP1***	0.88
	phoY1	0.87
σ^E	***sigB***	0.89
	hsp20	0.69

Supplementary Table S8. List of oligonuclotides used for ChIP-qPCR assay

Name of the primer	Sequence (5' → 3')
CHIP -140 *sigA* FW	GGACGCCCGTCGTCGC
CHIP -140 *sigA* RV	GACCACCGACGCCATCGC
CHIP -28 *sigA* FW	CAGCAACTGTGATCCGGTGT
CHIP -28 *sigA* RV	CTCACCGCTATTCGGTAGGC
CHIP +10 *sigA* FW	GCGGTGACGTTACAATGGAC
CHIP +10 *sigA* RV	GTTGGCGTTGTTATCGGCTG
CHIP +30 *sigA* FW	CGCGGTGACGTTACAATGGA
CHIP +30 *sigA* RV	CTTCACCGGAAGCGTCTGCTAA
CHIP +45 *sigA* FW	GCCTACCGAATAGCGGTGAGT
CHIP +45 *sigA* RV	CTTCACCGGAAGCGTCTGCTAA
CHIP +70 *sigA* FW	CAGCCGATAACAACGCCAAC
CHIP +70 *sigA* RV	CGGTCTTACGAATTCCCGGT
CHIP +150 *sigA* FW	AATTCGTAAGACCGAAAGGGTGTACG
CHIP +150 *sigA* RV	CCTTCTTGGCGGGGGTCTT
CHIP +500 *sigA* FW	CGCCCGCCGTCGCGA
CHIP +500 *sigA* RV	GGACGCCTTGTCCTTCTCGGACG
CHIP +700 *sigA* FW	GCCGAAGAAGAGGTGGAGCTGG
CHIP +700 *sigA* RV	AGTTCGGCCAGCTTCTGCGTG
CHIP +1375 *sigA* FW	GTGAGCTGCTGCAGGACCTGG
CHIP +1375 *sigA* RV	GGCGTGATGTCCATCTCCTTGGC
CHIP +1500 *sigA* FW	CAAGACCATGTCGAAGCTGCGCCA
CHIP +1500 *sigA* RV	CTAGTCCAGGTAGTCGCGCAGCAC
CHIP -160 *rrnA* FW	CGAGAGAGTAAGCTCGTAGGAAGCAAGACCC
CHIP -160 *rrnA* RV	GAGGCGGTTTGTGACGGATGGGA
CHIP -28 *rrnA* FW	GAGCGGAGAAAACAACCCGG
CHIP -28 *rrnA* RV	TTTTGGGGCAACCCTGCC
CHIP +10 *rrnA* FW	AGACAAAGCAGTATTAAGCTGGCAG
CHIP +10 *rrnA* RV	CAAACAACAGACCAGATTGTTAGCCG
CHIP +30 *rrnA* FW	GCAGGGTTGCCCCAAAACGG
CHIP +30 *rrnA* RV	ACCAAACACACTATTGAGTTCTCAAACAACAGACCAG
CHIP +45 *rrnA* FW	CAAAACGGGGCGGCTAACAAT
CHIP +45 *rrnA* RV	AAAAACAACAAACAAAAACCACCAAACACACTATTGAGTT
CHIP +70 *rrnA* FW	TGGTCTGTTGTTTGAGAACTCAATAGTGTGTTTG
CHIP +70 *rrnA* RV	CTAAACGGGAAAAAGAGGCGGACAAAAAA
CHIP +150 *rrnA* FW	GGGTGGATGTTTTTGATGCCAGTTTT
CHIP +150 *rrnA* RV	TCCAAAGACAGGTGAATTCACAATCAGAGAAAAT
CHIP +450 *rrnA* FW	CTTTTGCGGTGTGGGATGG
CHIP +450 *rrnA* RV	TACCCGTCGTCGCCTTG
CHIP +900 *rrnA* FW	GCGGTGGAATGCGCAGATATCAGGAG
CHIP +900 *rrnA* RV	CAGCGTCAGTTACTGCCCAGAGACC
CHIP +1020 *rrnA* FW	CGCCGTAAACGGTGGGTACTAGGTGT
CHIP +1020 *rrnA* RV	TACTTAATGCGTTAGCTACGGCACGGATCCC
CHIP +1220 *rrnA* FW	CGCCGGCAGAGATGTCGGTTC
CHIP +1220 *rrnA* RV	ACGACACGAGCTGACGACAGCC
CHIP +1510 *rrnA* FW	CTCAGTTCGGATCGGGGTCTGCAAC
CHIP +1510 *rrnA* RV	CGTTGCTGATCTGCGATTACTAGCGACTCC
CHIP +1620 *rrnA* FW	GTCACGTCATGAAAGTCGGTAACACCCGAAG
CHIP +1620 *rrnA* RV	CCACCTTCGACGGCTCCCTC
CHIP -131 *sigB* FW	CACCGCAACCGGGGACAG
CHIP -131 *sigB* RV	CCTGAGAATAGCGACAGAAGCCTCTCAG
CHIP -28 *sigB* FW	CAGGTCAACACGTATGAAAACGACGGCA
CHIP -28 *sigB* RV	GGCTCGAACTGTCATGACTTACAACGTGTCAT
CHIP +10 *sigB* FW	TCTTCGGGGGCATGACACGTTGTA

CHIP +10 *sigB* RV	GGCTTGTGGTGGCATTTGCCATGA
CHIP +45 *sigB* FW	GTTCGAGCCGATCAGGAGGCC
CHIP +45 *sigB* RV	TGCCGGGCTTTGGGCATC
CHIP +70 *sigB* FW	TGGCAAATGCCACCACAAGCCG
CHIP +70 *sigB* RV	CAGATACACGCGCACGAGGTCTG
CHIP +150 *sigB* FW	AACGGCATCGGCAAGACCGCATT
CHIP +150 *sigB* RV	GCGTACAACCCGGCCTCGAT
CHIP +450 *sigB* FW	TACACCAAGGGCTTCAAGTTCTCCACGTAC
CHIP +450 *sigB* RV	GCTCTGG2TCGGCCATGCC
CHIP +750 *sigB* FW	CATCGAGGATTCCGAGGCCATGTCC
CHIP +750 *sigB* RV	CGTAACGGATGTCGGTGTGCAGAAGC
CHIP +950 *sigB* FW	AGATCGAGCGCGAGGTCATGGC
CHIP +950 *sigB* RV	GCTCGCGTAGGAGCGGAGG
CHIP -140 *usfY* FW	TCGTTGTCCCAGCGCAACAGGTATC
CHIP -140 *usfY* RV	CTCGGTACCGCGAGCCAGG
CHIP -28 *usfY* FW	CGTCGACACTACGGCGAGCG
CHIP -28 *usfY* RV	CCGGGCAGGCCGGACTT
CHIP +30 *usfY* FW	TAGCCCGTAGGCAAGTCCGGC
CHIP +30 *usfY* RV	TCGCCCATTTACTGCTCCCTTCGG
CHIP +70 *usfY* FW	GATGATTCTGGGCGCCGCCGAA
CHIP +70 *usfY* RV	CCTTGTCGTTCGTGCATGGTCCACG
CHIP +200 *usfY* FW	TTGTTCCTGGGTGGTGTGTCCTTCGT
CHIP +200 *usfY* RV	ATGCCGACCGTCTCGTGGC
CHIP +350 *usfY* FW	AAGGAACGTCAATGGAATGCCGCTCAC
CHIP +350 *usfY* RV	CGAGTGACAACGCTCGGTCAGG
CHIP +450 *usfY* FW	GAAGCCGCCGCGCAGTATGAC
CHIP +450 *usfY* RV	GGACGTTCGTGGTCGAGTGGG
CHIP -145 *hsp20* FW	CCAACCCGTGCTGACCATCTGCT
CHIP -145 *hsp20* RV	CGCACGCCGTTGTCCACG
CHIP -28 *hsp20* FW	ATGAGCGTGGTCGACTCAAGTTATGAGAGGTGA
CHIP -28 *hsp20* RV	TCTGTCACCTCTCATAACTTGAGTCGACCACG
CHIP +10 *hsp20* FW	GGTCGACTCAAGTTATGAGAGGTGACAGACCG
CHIP +10 *hsp20* RV	CCCTTTCTCGTCTTCTCGACCGGC
CHIP +45 *hsp20* FW	CGGGGCCGGTCGAGAAGAC
CHIP +45 *hsp20* RV	CACCGCGGGCGTCTTCATCAG
CHIP +70 *hsp20* FW	GGGAGGCACAACGATGAGCACG
CHIP +70 *hsp20* RV	GAGCTGCCAGGCGGGC
CHIP +150 *hsp20* FW	GGACTTCTTCGGCCCGGC
CHIP +150 *hsp20* RV	TCTCTTGTGACCTCGGCGGC
CHIP +350 *hsp20* FW	CGGCCGCACCTACAGTGAGG
CHIP +350 *hsp20* RV	CCGTGACGTGCGCGGG
CHIP +500 *hsp20* FW	GCGCATCGCGATCGAGAGCC
CHIP +500 *hsp20* RV	GGTGCCACCGGTTCTTTCGGATC
CHIP -154 *phoU* FW	GCCCAGGGCGAGCCG
CHIP -154 *phoU* RV	GGAGCGTCGGCGGGC
CHIP -28 *phoU* FW	GATTGAAGGGCCCCTCGGATGG
CHIP -28 *phoU* RV	TGACCTCCCGCCCGG
CHIP +30 *phoU* FW	GGGCGGGAGGTCATGGG
CHIP +30 *phoU* RV	GCTCCGCGCTCAGCGC
CHIP +45 *phoU* FW	GGCGATGCGAGCTGTGTTCCAC
CHIP +45 *phoU* RV	CGCACATCTCGCCCAGCTCC
CHIP +70 *phoU* FW	CAGCTCACCGCGCTGAGC
CHIP +70 *phoU* RV	CGCTCCATCGCGCTCGC
CHIP +200 *phoU* FW	TCATCGCGCTGCGCAACC
CHIP +200 *phoU* RV	GGCCACGGGCTGCTG
CHIP +350 *phoU* FW	CCCGCATCTCCCGGCTG

CHIP +350 *phoU* RV	CATGGCGGCGATGCTGGC
CHIP +650 *phoU* FW	GCAAGCGCGTGGTGTTCGA
CHIP +650 *phoU* RV	GCTAGCCGGTAATCAGGCGAGTTTCT

Supplementary Table S9. Promoter-like motifs on downstream gene sequences used in ChIP assay

σ Factor	*Msm* Gene	Promoter-Like Motif	Position Post TSS	Consensus/Promoter Sequences Searched in Genes
σ^A	*16S rrnA*	**σ^A -35 like (+ strand)**	2, 37, 41, 71, 78, 85, 145, 168, 195, 201, 214, 564, 623, 728, 775, 1170, 1292, 1302, 1570	-35 consensus: TTGHNH<br>-10 consensus: TANNNT
		**σ^A -35 like (- strand)**	27, 52, 261, 571, 580, 655, 1093, 1117, 1148, 1288, 1348, 1429, 1467, 1684	
		**σ^A -10 like (+ strand)**	53, 715, 829, 970, 1314, 1413, 1556	
		**σ^A -10 (- strand)**	664, 798, 830, 835, 1085, 1162, 1315, 1414, 1688	
		**NusA (*nut* sites)**	2-63 bp	G**TTGCCCCAAAACGGGGCGG**CTAACAATCTGGTC**TGTTGTTTGAGAA**CTCAAT**AGTGTGTTT**G
		**NusG**	37, 71, 78, 81, 195, 201	TTNTTT
	sigA	**σ^A -35 like (- strand)**	46, 143, 528, 588, 663, 912, 1068	-35 consensus: TTGHNH<br>-10 consensus: TANNNT
σ^F	*usfY*	**σ^F -35 like (+ strand)**	327	-35 elements: "GGGTTT", "GGATTG", "CGGTCA", "CGGTCA", "CGGTTT", "AAGTTT", "TGGAAT", "TGGAAG"<br>-10 elements: "GAACAT", "GGGTAC", "GGGCAT", "GGGTAT", "CGATAG", "GGGTAG", "GGGTTC"
		**σ^A -35 like (+ strand)**	165	-35 consensus: TTGHNH<br>-10 consensus: TANNNT
	phoU	**σ^F-35 like (+ strand)**	117	-35 elemets: "GGGTTT", "GGATTG", "CGGTCA", "CGGTCA", "CGGTTT", "AAGTTT", "TGGAAT", "TGGAAG"<br>-10 elements: "GAACAT", "GGGTAC", "GGGCAT", "GGGTAT", "CGATAG", "GGGTAG", "GGGTTC"
		**σ^A -35 like (-strand)**	180, 207, 421 and 314	-35 consensus: TTGHNH<br>-10 consensus: TANNNT
		**σ^A -10 like (+ strand)**	247	
σ^E	*sigB*	**σ^E -35 like (+ strand)**	371, 592	-35 elements: "GGAATA", "ATAGCG", "GGAACA", "GGAAAT", "GGGAAC", "GGAACT"<br>-10 elements: "CGTTG", "GGTTG", "CGTTA", "AGTTA", "GGTTC"
		**σ^E-10 like (+ strand)**	52, 180, 348, 782, 834	
		**σ^E-10 like (- strand)**	299, 377, 569, 635, 688, 950	
		**σ^A -35 like (+ strand)**	54, 131, 182, 350, 642	-35 consensus: TTGHNH<br>-10 consensus: TANNNT
		**σ^A -35 like (- strand)**	35, 138, 234, 531, 633	
	hsp20	**σ^E-10 like (+ strand)**	146, 266	-35 elements: "GGAATA", "ATAGCG", "GGAACA", "GGAAAT", "GGGAAC", "GGAACT"<br>-10 elements: "CGTTG", "GGTTG", "CGTTA", "AGTTA", "GGTTC"
		**σ^E-10 like (- strand)**	51, 246	
		**σ^A -10 like (+ strand)**	325	-35 consensus: TTGHNH<br>-10 consensus: TANNNT
		**σ^A -10 like (-strand)**	429	
		**σ^A -35 like (- strand)**	49, 152, 181, 365	

Supplementary Table S10. Sequences used for EMSA assay to show binding of non-cognate RNAP on intragenic regions in Mycobacteria

Gene	Position	Sequence (5’ → 3’)
Msm sigB	+53	CCGTCATGGCAAATGCCACCACAAGCCGCGTTGACACCGA TCTGGATGCCCAAAGCCCGG
	+130	GTGTATCTGAACGGCATCGGCAAGACCGCATTGCTCAATGC CGCAGACGAAGTAGAACTCGC
	+280	GTGGTCCGCGACGGCGAGGCGGCACGGCGACACCTGCTC GAGGCCAACCTGCGCCTCGTG
	+349	GCCAAGCGCTACACCGGTCGCGGGATGCCGTTGCTCGACC TGATCCAGGAGGGGAACCTGG
Msm hsp20	+48	CGAGAAGACGAGAAAGGGAGGCACAACGATGAGCACGCTG ATGAAGACG
	+151	TTCGGCCCGGCCGACGACTGGTTCAAGGGATTCACCCCGG CCGCCGAGGT
	+465	TGCCTACGCGGGCGAGCAGCCTCAGCGCATCGCGATCGAG AGCCGGTAA
Mtb sigB	-	CTGCACGTCACAGGGCGTCAGATCACTGCTGGGTGGGAACT CAAAGTCCGGCTTTGTCGTTAAACCCCATGACAGTGCAAGCC GATCGGGAGGTCGCTATGGCCGATGCACCCACAAGGGCCACC ACAAGCCGGGTTGACAGCGATCTGGAT

Supplementary References

1. Banerjee,R., Rudra,P., Prajapati,R.K., Sengupta,S. and Mukhopadhyay,J. (2014) Optimization of recombinant Mycobacterium tuberculosis RNA polymerase expression and purification. *Tuberculosis (Edinb).*, **94**, 397–404.
2. Chin,J.W., Santoro,S.W., Martin,A.B., King,D.S., Wang,L. and Schultz,P.G. (2002) Addition of p-azido-L-phenylalanine to the genetic code of Escherichia coli. *J. Am. Chem. Soc.*, **124**, 9026–9027.
3. Jacques,J.-F., Rodrigue,S., Brzezinski,R. and Gaudreau,L. (2006) A recombinant Mycobacterium tuberculosis in vitro transcription system. *FEMS Microbiol. Lett.*, **255**, 140–147.
